# Topological data analysis in biomedicine: A review

**DOI:** 10.1016/j.jbi.2022.104082

**Published:** 2022-05-01

**Authors:** Yara Skaf, Reinhard Laubenbacher

**Affiliations:** a University of Florida, Department of Mathematics, Gainesville, FL, USA; b University of Florida, Department of Medicine, Division of Pulmonary, Critical Care, & Sleep Medicine, Gainesville, FL, USA

**Keywords:** Biomedical informatics, Personalized medicine, Big data analytics, Topological data analysis, TDA, Applied topology, Mapper, Persistent homology, Machine learning

## Abstract

Significant technological advances made in recent years have shepherded a dramatic increase in utilization of digital technologies for biomedicine– everything from the widespread use of electronic health records to improved medical imaging capabilities and the rising ubiquity of genomic sequencing contribute to a “digitization” of biomedical research and clinical care. With this shift toward computerized tools comes a dramatic increase in the amount of available data, and current tools for data analysis capable of extracting meaningful knowledge from this wealth of information have yet to catch up. This article seeks to provide an overview of emerging mathematical methods with the potential to improve the abilities of clinicians and researchers to analyze biomedical data, but may be hindered from doing so by a lack of conceptual accessibility and awareness in the life sciences research community. In particular, we focus on *topological data analysis (TDA)*, a set of methods grounded in the mathematical field of algebraic topology that seeks to describe and harness features related to the “shape” of data. We aim to make such techniques more approachable to non-mathematicians by providing a conceptual discussion of their theoretical foundations followed by a survey of their published applications to scientific research. Finally, we discuss the limitations of these methods and suggest potential avenues for future work integrating mathematical tools into clinical care and biomedical informatics.

## 1. Introduction

The escalating development of digital technologies over the last several decades has given rise to an accompanying surge in available data. For this vast amount of information to be useful, it must be processed and analyzed to extract meaning that may be obscured in its raw form. However, its size and complexity often exceeds the capabilities of traditional tools, motivating the ongoing research effort to develop novel analytic techniques better able to cope with these challenges. This effort has resulted in the maturation of disciplines such as artificial intelligence, machine learning (ML), data mining, parallel computing, and many others. Despite these advances, innumerable challenges of modern informatics remain unaddressed and strategies for extracting insight from complex data will undoubtedly persist as an area of active research for years to come.

The emerging field of *topological data analysis (TDA)* provides a number of potential avenues for addressing some of these issues. TDA encompasses a set of tools for data visualization, exploration, and analysis that are grounded in *topology*, an area of mathematics that studies abstract notions of shape and connectivity. By focusing on the “shape” of data, TDA takes a fundamentally different perspective than other approaches, which has proven effective at detecting features that are missed by these methods. Unfortunately, the rigorous theoretical foundation that is responsible for the power of TDA also makes it less approachable to researchers outside pure math. As such, TDA enjoys far less notoriety than more easily usable or marketable methods like artificial intelligence, and applications to research in the life sciences remain limited.

In this review, we aim to provide an introduction to TDA that is accessible to biomedical researchers or data scientists with minimal training in theoretical math. We begin by describing the unique challenges presented by modern data analysis and ways in which traditional techniques fail to address them, necessitating the development of alternative approaches. We then describe the conceptual underpinnings of the major TDA algorithms along with some of their practical applications. We hope that the long list of examples included in Section 4 and summarized in Table 1 can serve as a useful reference detailing published applications of TDA to biomedicine. To conclude, we discuss advantages and limitations of TDA as compared to standard tools and propose some potential avenues for future research and development in this field.

### 1.1. Guide for readers

Since the full article is quite lengthy, certain readers may want to restrict their attention to sections most relevant to their interests. For those looking for a quick, high-level overview of TDA and its biomedical applications, we would recommend focusing on the figures and associated text in Section 3 as well as the summaries provided in Tables 1 & 2 and Fig. 14. Readers interested in understanding the mechanics of TDA algorithms may want to read Section 3 in detail, while those interested in their benefits relative to other tools could focus on Sections 2 & 5. Those who are generally familiar with TDA but want to learn more about its applications can safely skip Sections 2 & 3 and instead focus on Section 4. Finally, anyone hoping to learn how to apply TDA to their own work might benefit from reading the article in its entirety, focusing in particular on Sections 5 & 6.

## 2. Background: Big data analytics

### 2.1. “Big Data” in biomedicine

Technological advances have brought about a “digitization” of biomedical research and clinical care. In any modern hospital, every encounter with a patient is documented in an electronic health record that includes information such as vital signs, lab values, images, and much more. Next-generation sequencing technologies have become affordable and ubiquitous, adding genomics data to this wealth of information. Data generated by decades of scientific research is now curated in vast databases of DNA sequences, single nucleotide polymorphisms, and protein structures that are readily available for new analysis. Further, the quantity and complexity of this data multiplies every day as more information is produced, and the modern researcher has all of this at their fingertips.

In biomedicine, we ideally could use this information to better understand mechanisms of disease, predict clinical outcomes, or design optimal treatments to improve patient care. However, this is impossible without analytic tools capable of extracting meaningful knowledge from the huge amount of available data. This idea is intrinsic to what is referred to as the problem of “big data”, meaning data of a size or complexity beyond the ability of traditional methods and software to store, process, and analyze. The challenges of working with such information are summarized by the “Four V’s of Big Data”: [85,56,103,46,86].

1. Volume refers to quantity, one facet of which is the often large number of data points within a data set. The other is dimensionality, meaning the number of variables present for each data point. Both of these contribute significantly to computational limitations encountered with large data sets.
2. Velocity describes the often rapid or continuous rate at which data is generated, compounding issues of volume.
3. Variety deals with the nature of data. Modern data is frequently extremely heterogeneous, meaning it may vary widely in type, structure, content, and format, making it difficult to process consistently for use in many algorithms.
4. Veracity refers to quality and reliability. To be useful, data must be sufficiently reflective of whatever real-world phenomenon they describe– if a data set is too noisy, skewed, incomplete, or otherwise low quality, no amount of analysis will be able to extract meaningful insight.

### 2.2. Limitations of standard analytic tools

Even the most cutting-edge computational tools are imperfect in their ability to handle big data. Common analytic approaches in biomedicine include classical statistics, network analysis, ordination, clustering, and machine learning. Each of these is equipped to answer different types of questions and comes with its own set of limitations. Here we discuss certain drawbacks of such methods, many of which are addressed by TDA.

#### 2.2.1. Detection of multivariate shape-based features

Though statistics certainly have an important place in any data analysis pipeline, they frequently require hypothesis in advance of analysis are fundamentally limited in their ability to detect certain features in data. This is illustrated by the “Datasaurus” [95], which includes 13 very different data sets that have the exact same summary statistics (see Fig. 1). Of course, there are many other ways to distinguish the data sets in this simple example. However, as we will see in Section 3, TDA is a natural framework to identify and characterize these types of differences without knowing whether they exist in advance.

Standard tools also struggle to detect and encode higher-dimensional features in big data. For example, clustering or ordination methods such as principle components analysis (PCA) are frequently used for exploratory analysis of high-dimensional data. While useful for visualizing certain patterns, these tools may fail to capture multivariate features. Network approaches are reasonably effective at representing and characterizing relationships between species in a system. However, while generally able to describe pairwise interactions, such methods can be limited in their ability to capture group dynamics or higher order interactions between more than two species.

#### 2.2.2. Local & global behavior

A common limitation among traditional computational tools is *local variation*, meaning variable performance across subgroups. [79] For instance, population-level statistics are the basis for many ubiquitous clinical tools such as risk scores for predicting adverse outcomes from a set of easily-measured physiologic variables. However, these scores may be highly inaccurate for patients who differ significantly from the population average upon which the tool was built. Even more complex models are generally fitted only across entire populations– standard error minimization methods implicitly seek to optimize performance across the full data set, disregarding subsets on which performance is much lower than the mean. This can lead to erroneous predictions within subpopulations on which a model performs poorly. To visualize this, consider the simple example in Fig. 1. The average value for any of these data sets is somewhere around the middle of the plot. However, this value is not representative of most (if any) of the data points. If these data encoded patient physiology, a treatment targeted at this average value would be appropriate *globally* for the population “on average” but not *locally* for any individual patient.

On the other hand, tools such as clustering are effective at making individual predictions or detecting local subgroups of similar data, but provide minimal insight into how individuals or subgroups relate to form the overall population. In other words, some models are globally accurate but may be locally unreliable while others are sensitive to local information but fail to detect global structure. TDA will provide a unified framework that cohesively incorporates both types of information.

#### 2.2.3. Interpretability & parameter choices

Interpretability is an important consideration for computational models– an ideal model should be both *explainable*, meaning its internal workings and decision-making process should be straightforward to describe in human terms; and *interpretable*, so that its actions are understandable to users. This is a frequent topic of discourse in ML, where many tools are ”black box models” that are difficult to explore or inspect. [71,127] While such models may be highly accurate and even outperform humans, studies have shown that most users do not trust a model unless they can understand its predictions and evaluate whether its reasoning is sound. [14,50].

On a related note, many common algorithms depend on a number of parameters that can be chosen arbitrarily by the end user. These choices can significantly impact the quality of results, but the average scientific researcher may lack the technical background to fully understand how every parameter affects the behavior of the model or how to select and optimize them appropriately. It is therefore virtually impossible for many users to make intelligent choices about their values, especially for models that are not very interpretable.

While TDA does not fully eliminate either of these problems, it provides a number of improvements to interpretability and stability with respect to small changes in input data or parameters.

#### 2.2.4. Sample sizes, training, & bias

Statistical methods often require large sample sizes to draw conclusions about the distribution of a data set or ensure adequate power to extract significant features. ML models may require exceedingly large data sets for training that may be impractical to acquire in certain contexts. For example, implementing a ML classifier to diagnose tumor subtypes based on histology would require thousands of pre-labelled images to train the model, and it may not always be possible to obtain that quantity of pre-labelled data. Further, ML tools are prone to issues such as bias and over-parametrization that adversely impact their ability to generalize their learned predictions to new data. Regularization techniques can mitigate this to some degree, but their efficacy on big data sets is not entirely clear. [32,68,23,60].

#### 2.2.5. Dimensionality

The “curse of dimensionality” refers to the fact that many algorithms with high accuracy on low-dimensional data exhibit significantly worse performance on high-dimensional input. In particular for ML models, the Hughes effect states that the predictive ability of the model decreases as dimensionality of the training data increases. A major contributor to this curse is the fact that similarity measures become somewhat less effective in high dimensions– data points that differ significantly in one variable will clearly be measured as distinct in three dimensions, but may appear almost identical in 100 dimensions if they have similar values for the other 99 variables. Another contributor for ML models is the increased difficulty of feature engineering and feature selection in high-dimensional spaces. [86].

A number of dimensionality reduction techniques have been developed to address challenges related to the overwhelmingly large size of biomedical data, but these are far from optimal in many situations. First, these algorithms are generally quite computationally intensive on large data sets. Even more importantly, there is no clear solution to the problem of developing or identifying algorithms that are both computationally efficient and accurate in terms of their ability to reduce dimensionality in such a way as to highlight features within the reduced data that were obscured in the high-dimensional data. TDA tools that produce low-dimensional topological summaries of data are one possible approach to addressing these challenges. [90].

#### 2.2.6. Noise & non-linearity

Many metrics for evaluating data such as correlation coefficients rely on implicit or explicit assumptions of linearity, and studies have shown that the performance of neural networks and other ML models is severely adversely affected by non-linearity of input data. Moreover, as well as generally being more pronounced in big data, non-linearity is much more difficult to evaluate in these scenarios where data is too high-dimensional or complex for linearity to be assessed visually. Data quality issues such as noise can also significantly impact results, in particular for statistical tools and ML model training. While data quality will necessarily impact any form of analysis, TDA has proven to be more robust to certain types of noise and better able to detect non-linear relationships than most other approaches.

## 3. Introduction to TDA

*Topological data analysis (TDA)* provides a number of potential strategies for addressing the limitations described in the previous section. We first provide a brief introduction to the key ideas from topology that form a basis for this set of methods, then go on to outline the algorithms themselves.

### 3.1. What is topology?

*Topology* is a branch of mathematics focused on abstract notions of shape and continuity, primarily concerned with properties that are preserved after *continuous deformation*– objects are topologically equivalent if one can be transformed into the other via continuous operations such as stretching, bending, or twisting but not tearing, gluing, or self-intersecting (Fig. 2).

A critical feature of this definition is the preservation of some concept of “nearness”. Points can only get farther apart as part of a *continuous* transformation– nearby points cannot suddenly become distant along a transition to a topologically equivalent space. For example, the two points highlighted in Fig. 2C are near on the coffee cup and they remain near at every step. If the handle of the cup were to be broken off, however, that would separate the points suddenly in a non-continuous fashion and would break the topological equivalence. This idea is integral to certain advantageous properties of TDA such as its ability to be coordinate-free. These are discussed further in Section 6.

### 3.2. TDA algorithms

The two main TDA methods are *Mapper* and *persistent homology*. ^1^ We begin with a brief overview of the concepts underpinning persistent homology, then go on to describe Mapper. While the formal theory of homology is too complex to address in detail here, we aim to provide a simplified, intuitive introduction that should suffice to understand why the idea is useful for data analysis. For more rigorous discussions, see [67,25,63,96,54,108].

#### 3.2.1. Persistent homology

According to the Oxford English Dictionary, objects are *homologous* if they have “the same relation, proportion, relative position, etc.”, implying some vague sense of similarity between them. Though the mathematical definition is somewhat more precise, it remains true to this general concept.

Homology uses theoretical tools from abstract algebra to detect topologically interesting features such as connected components or higher-dimensional holes, and homologous objects will share the same features. Though it is possible for different objects to have the same homology, spaces with non-equivalent homology *must* be topologically distinct. Homology therefore provides a rigorous way of proving objects are in fact not the same, no matter how similar they may appear from other perspectives.

More specifically, homology counts the topological features in each dimension. In dimension 0, this corresponds to the number of connected components, which is denoted by *β*_0_. Points are homologous if they belong to the same connected component.

**Table:** 

*β*_0_ = 0	*β*_0_ = 1	*β*_0_ = 2
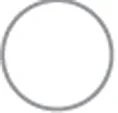	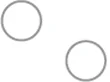	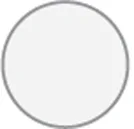

Note that no nonempty space can have zero connected components, so there is no example with *β*_0_ = 0.

In 1D, *β*_1_ corresponds to the number of *unique* 1-dimensional holes, meaning loops or circles that are not filled in.

**Table:** 

*β*_1_ = 0	*β*_1_ = 1	*β*_1_ = 2
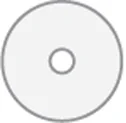	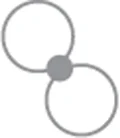	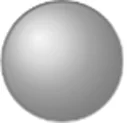

Though it may seem that these should be 2-dimensional since they appear to be 2D regions of space, the feature itself includes only the 1D curve that forms the *boundary* of the hole, so it is considered 1D. Also notice the word unique in this definition– if one loop can be continuously deformed into another, they are topologically the same and thus counted as a single 1D hole.

Analogously, *β*_2_ counts unique 2-dimensional holes, meaning 3-dimensional voids or cavities bounded by 2D surfaces.

**Table:** 

*β*_2_ = 0	*β*_2_ = 1	*β*_2_ = 2
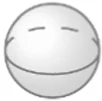	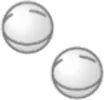	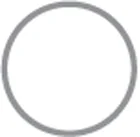

In general, *β*_*n*_ is called the *n-th Betti number* and is defined as the number of *n*-dimensional holes in a topological space. In this way, homology can distinguish shapes such as circles, spheres, solid balls, and donuts based on their Betti numbers:

While homology alone cannot distinguish every possible shape (e.g. a 2D solid disk has the same Betti numbers as a 1D line segment), it still provides a concise description of useful topological information in these situations.

**Table:** 

	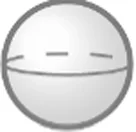	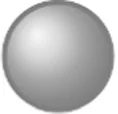	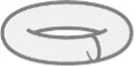	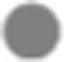 0-simplex	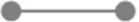 1-simplex		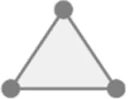 2-simplex	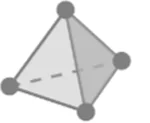 3-simplex

*β*_0_	1	1	1			1		
*β*_1_	1	0	0			2		
*β*_2_	0	1	0			1		

The *persistence* piece of persistent homology is best explained by an example. Suppose the data set we wish to analyze is that shown in Fig. 3. We can observe that these data lie roughly in the shape of a circle. Persistent homology will allow us to draw the same conclusion using mathematical tools alone, which will be useful when data is too complex to examine visually.

The general idea of the procedure is to connect points that are relatively close together, then examine the homology of the resulting shape. The number of connections added at each step will depend on a parameter dictating how close points should be to get connected, and it is not always clear how to choose this value so that the output of the algorithm is an optimal representation of the input data. To get around this subjective choice, we can repeat the procedure for various definitions of “relatively close together”, meaning across a range of scales.

To begin, consider the input data on the far left in Fig. 3. In this panel, each data point lies within its own connected component containing only that point, so *β*_0_ = # connected components = # points = 8. This is represented by the eight different 0-dimensional features depicted by the blue lines below the data, and we say that the *birth* of these features occurs at value 0 of the scale parameter. Next, we construct balls of some small radius around each point in the data set, connecting points if and only if their balls intersect. In the second panel of Fig. 3, each point is the center of its own ball and the two highlighted points at the top right are connected by an edge since their balls overlap. These two points are now in the same connected component, meaning there are now 7 components instead of 8. This *death* of a 0D feature is shown by the line representing one of the components terminating at this scale value. We continue this procedure for larger and larger balls, recording any changes to the homology as the radius increases.

Also notice that the only 1D feature is born in the second to last panel of Fig. 3 when the radius is large enough that the edges between points connect to form a loop, and the feature dies in the last panel when the radius is large enough that this loop is completely filled in by the balls. Detection of features in higher dimensions (e.g. voids, cavities) proceeds analogously.

The network created at each step is called a *simplicial complex*, a generalized type of graph that is discussed further in Section 3.2.2. Unlike standard networks, simplicial complexes can contain higher-dimensional analogs of edges between more than two points, allowing them to represent higher-dimensional homology. There are many ways to construct simplicial complexes on a data set, but the procedure described above produces a sequence of *Vietoris-Rips complexes*. The diagram that records the homology in the bottom of Fig. 3 is called a *persistence barcode*, in which each bar begins at some feature’s birth and ends at its death. The same information can also be represented as a graph in the Euclidean plane where (*x*, *y*) = (*birth*, *death*). This representation is called a *persistence diagram*.

#### 3.2.2. Mapper

*Mapper* [136] is another TDA tool that has proven particularly useful for data visualization and exploration. This algorithm distills information from high-dimensional data into a simple topological skeleton in the form of a simplicial complex, which is useful for big data that are too complicated to be easily examined visually or statistically. The steps are described below, both with respect to the simple example shown in Fig. 4 and in the context of a potential real-world application.

1. Input: **a** *point cloud* ***X***, meaning any finite set of data points. In Fig. 4A, *X* is a set of points roughly gathered around a circle. However, *X* need not be the type of data that can easily be plotted on a plane– in our example application, take *X* to be a data set sourced from an electronic health record (EHR), consisting of patients represented as “phenotype vectors” of baseline clinical variables (e.g. vital signs, lab values, etc.).
2. Lens: **Apply** *lens function* ***f*** : ***X***⟶***Z*** **to each point in** ***X***. This key step maps data from *X* into a *lensed space* *Z*, which should generally be simpler than *X* in some way (e.g. lower dimension, more computable metric) so that generating a cover in the next step is computationally and conceptually tractable. Further, the function *f* should be chosen to reflect a feature of interest– different lenses retain different types of information, and this information will be visually represented and used to group data in the final output. In this way, *f* acts as a “lens” through which data is viewed.
  The lensed space is frequently chosen to be *Z* = ℝ, meaning the lens simply associates a value to each data point in the form of a single number. Common examples include local density estimation (e.g. Gaussian kernel), eccentricity/centrality, and graph Laplacians. These functions all reflect geometric properties, which is useful for studying the spatial arrangement of the underlying data. In general, the lens function could take any number of forms. Using projection values from PCA as a lens can provide a compressed and refined representation of PCA output, which is often useful for large data sets.
  Custom lenses can also be formulated to reflect domain-specific properties such as the color of a pixel in an image or the value of a clinical risk score for a patient. For example, probability values obtained from ML models can also be used as lens functions, which allows for visual exploration of data in terms of how it is classified by the more opaque ML model. In contrast to functions such as eccentricity and density that provide information about geometric features of the data, these types of lenses yield insight into how these intrinsic features of the underlying data interact with interesting properties encoded by the lens. Further, lenses need not be 1D– maps into higher-dimensional spaces can give higher-dimensional complexes as output. [136].
  In Fig. 4B, we may be interested in the spatial arrangement of the data, so each point of *X* is mapped to its *x*-coordinate by *f*. In our EHR example, we will use the lens to encode mortality risk, a clinically interesting property we plan to study.
3. Cover: **Construct a cover of the lensed data** *f*(*X*) by dividing it into overlapping subsets or “bins”. For the noisy circle, this corresponds to the overlapping intervals in Fig. 4C. For the clinical data lensed by mortality risk, an analogous cover would correspond to overlapping risk levels or strata.
  It is important to note that this cover groups nearby data points in a way that is entirely *independent* of their similarity at baseline in the input space *X*. In Fig. 4, points in the same cover set may have similar *x*-coordinates but be far apart in *X* if they have very different *y*-coordinates. Similarly, two patients in the same cover set will have very similar mortality risk but they may be completely different in every other way, such as a patient suffering from a severe myocardial infarction and another with an intracranial hemorrhage. This allows the output of Mapper to represent similarity in two independent ways, both at baseline and in terms of some property or properties of interest.
4. Pullback: **Examine the subsets of** ***X* that are lensed into each individual cover set by** ***f***. These subsets correspond to the points highlighted in each rectangle shown in Fig. 4D. Note that points within the same rectangle may or may not be near each other, as discussed above. For EHR data, these correspond to all patients (not necessarily similar patients) that map into a certain range of the lens function (e.g. all patients with mortality risk between 0.9 and 1).
5. Cluster: **Perform clustering on data** *within each individual pullback set* (as opposed to clustering the entire data set at once). This gives the subgroups of nearby points within each colored rectangle in Fig. 4D (e.g. the two distinct clusters of points in the middle green rectangle). In our clinical example, these would be distinct clusters of phenotypically similar patients, organized by value of the lens (e.g. all *distinct phenotypic subtypes* of patients with mortality risk from 0.9–1).
6. Output: **Generate a simplicial complex** by representing clusters as nodes and connecting any nodes whose clusters overlap by an edge. In Fig. 4E, the output retains the 1D homological feature (the circle) that existed in the initial data, but presents it in a simplified form annotated by the value of the lens function. On the EHR data, this output represents a phenotypic similarity network of patients, annotated by mortality risk, where edges connect nodes representing similar or closely related phenotypes. Examining paths from low to high risk areas within the complex can therefore provide insight into processes such as progression from mild to severe disease states.

Note that if a higher-dimensional input data set or lens function was used, higher-dimensional overlaps between more than two clusters at once could occur. These would be encoded as *n-simplices*, which can be thought of analogs to edges generalized to any arbitrary number of dimensions.


N/A

### 3.3. Summary & implications

TDA tools take large, high-dimensional, complex data as input and output a simpler, low-dimensional representation that maintains topological properties related to shape and connectivity. The two main methods are persistent homology, which is useful for generating a quantitative representation of homological features across a range of scales that can serve as a topological fingerprint for the input data, and Mapper, which constructs a compact visual summary suitable for exploratory data analysis.

Note that, while the examples described above are relatively easy to understand, they do not depict the full power of TDA. In Figs. 3 and 4, we can easily see all of the topological structure of the data– there is one main component and points lie around a single loop. However, these types of observations are often impossible to make in real-world situations, especially with unwieldy biomedical data that are too complex to be easily plotted. TDA tools therefore allow us to generalize this process of detecting shapes and structures to data that are difficult to visually examine. Fig. 14 in the Discussion section attempts to illustrate this in a more realistic setting.

We will discuss the advantages and limitations of these methods further in Section 6, but first we examine some applications to gain a better sense for their practical utility.

## 4. Applications of TDA

Use of topology in fields outside of theoretical mathematics is on the rise– published applications ranging from hyperspectral imaging [53] to viral evolution [28] and analysis of financial markets [8] showcase the potential of TDA for research across a myriad of domains. We discuss a subset of this work pertaining to healthcare and biomedicine here. This information is also summarized in Table 1 at the end of the section.

### 4.1. Clinical care & precision medicine

Despite increasing efforts to consider individualized factors such as genomics and biomarkers, most medical care remains largely standardized– diseases and treatments are studied at a population level, and this information is extrapolated and assumed to apply to each individual. However, this is not always the case, especially for patients who differ significantly from the “average” patient. *Precision medicine* takes an alternative approach, seeking to tailor care to each individual based on the unique physiologic factors that distinguish them. ML tools have proven useful in this domain [99,33,92], but TDA provides another perspective that may be particularly helpful for multifactorial diseases and heterogeneous populations of complex patients.

#### 4.1.1. Patient subtyping

An important facet of precision medicine is subtyping, since different forms of a single disease may require radically different management. For example, diabetes mellitus has long been considered to have two major subtypes, each of which has completely different underlying causes, risk factors, and pathophysiology. It is widely accepted that these subtypes must be treated differently– for instance, type 1 diabetics should generally be managed with insulin while metformin is the standard initial pharmacologic intervention for type 2 diabetics. Tailoring care based on disease subtype results in substantially better outcomes than if type 1 and type 2 diabetes patients were treated identically. The challenge with this approach is that not all diseases have subtypes as well-characterized as diabetes, which presents an ongoing need for tools that can help elucidate such subtypes in complex pathophysiologic states and more finely stratify patients within broad diagnosis classes.

##### 4.1.1.1. Diabetes.

Extending the above example, type 2 diabetes mellitus (T2DM) is a complex pathology that affects a significant and increasing subset of the US population, meaning improved strategies for early prevention and treatment could have a major impact on healthcare. A growing body of literature suggests that the diagnosis of T2DM actually encapsulates a number of distinct disease subtypes, each of which may differ in clinical phenotype and response to therapeutic interventions. A precision medicine approach that accounts for such subtypes could therefore significantly improve care for T2DM patients.

To this end, Li et al. [87] used a TDA-based approach to to identify novel subgroups of T2DM patients in a high-dimensional data set comprised of genotype information and clinical variables taken from EHRs. The authors generated a Mapper network that successfully identified three distinct subgroups (Fig. 5). Subgroup 1 was characterized by known T2DM complications such as diabetic nephropathy and retinopathy, subgroup 2 by cancer and cardiovascular disease, and subgroup 3 by cardiovascular and neurological diseases, allergies, and HIV. These results were built upon and validated by subsequent genetic association analysis that demonstrated a number of genetic markers specific to each subtype, and that the functions of these genes aligned with the clinical phenotypes of each subgroup.

Diagliati et al. [45] used an alternate method relying on *temporal phenotypes*, or sequences of clinical events that occur over a period of time. Temporal phenotypes are useful for examining how a disease progresses and whether progression differs for different patient subgroups. The authors use a combination of Mapper and pseudo time-series analyses to identify and characterize disease trajectories corresponding to temporal phenotypes. Mapper revealed several distinct subtypes of T2DM, including one that differs significantly from the population average in terms of cholesterol and initial HbA1c levels.

##### 4.1.1.2. Aortic stenosis.

Mapper has also been used by Casaclang-Verzosa et al. to study aortic stenosis, where it was able to identify disease subtypes with a higher resolution than standard approaches. [27] The authors constructed a Mapper representation of 246 patients that had previously been diagnosed with aortic stenosis. Four echocardiographic variables (aortic valve area, left ventricular ejection fraction, left ventricular mass index, relative wall thickness) were taken from patient records and used as input for the algorithm. Based on this, two distinct pathways from mild to severe disease were identified (Fig. 6). The first consisted of patients whose valvular function decreased while ejection fraction was largely preserved throughout the course of disease progression, while the second was characterized by primarily valvular dysfunction with preserved ejection fraction. The existence of these distinct pathways suggests there are potentially different underlying pathophysiologic mechanisms governing the two disease subtypes.

Further, the authors noted that aortic valve replacement seems to move patients from the region of the network associated with severe disease states toward the mild or moderate disease areas, illustrating how Mapper can adapt to changes in the data brought about by administering treatments or other interventions. This allows the technique to easily be used to explore how various changes in a patient’s physiological phenotype will affect their position in the network, facilitating data-driven predictions about which interventions would be most effective to move that patient toward a less severe disease state.

##### 4.1.1.3. Asthma.

Asthma is a relatively common and potentially debilitating chronic condition that causes obstruction of airways in the lungs, leading to symptoms such as wheezing and shortness of breath. The disease is thought to be caused by some combination of genetic and environmental factors, but in general the pathophysiology is poorly understood. Studying this condition is made more complex by the fact that triggers, manifestations, and responses to treatment are highly variable. [94,17].

In an early application of topological tools to investigate this complex pathophysiology, Hinks et al. [69] examined immune cells and inflammatory mediators in an attempt to elucidate the relationships between these immunologic factors and asthma severity. Each asthmatic and healthy control patient was assessed via clinical examination and measurement of several types of immune cells, cytokines, and mediators in sputum, endobronchial biopsy, and bronchoalveolar lavage samples. When this information was used as input for Mapper, one subgroup of healthy patients and six subgroups of asthmatic patients were clearly identified. Further, each subgroup demonstrated distinct clinical and pathological characteristics that were easily visualized using the Mapper network (Fig. 7).

A more recent study by Siddiqui et al. [135] took a related approach to examining heterogeneity in asthma by investigating pathological airway changes as a means to more finely classify this diverse population of patients. The authors used Mapper to visualize previously validated pathological features of bronchial biopsy samples (e.g. basement membrane thickness, inflammatory cell counts, etc.) from asthmatic patients and healthy controls. Mapper identified four distinct subgroups of asthma patients, each of which had several “microclusters” that were missed by population-level statistics. Moreover, Mapper depicts the continuous transition from a relatively healthy state (TDA Group 1) to a mild/moderate disease state (TDA Group 2) by paths within the Mapper network between these two patient subgroups. Subsequent analysis of these subgroups revealed distinct pathological and clinical features characterizing each subtype, once again highlighting the use of Mapper as a supplement to clustering for identifying disease subgroups.

Finally, two transcriptomics-based studies of data from the U-BIO-PRED (Unbiased Biomarkers in Prediction of Respiratory Disease Outcomes) project also demonstrated the utility of TDA for identifying asthma subtypes. Kuo et al. [80] applied Mapper to clinical and histologic data from bronchial biopsies and endothelial brushings from asthma patients. Mapper identified two distinct subtypes, one characterized by high expression levels for T_*H*_2 cell cytokines and the other by a lack of response to corticosteroid treatment. In a different study, Bigler et al. [13] showed how transcriptomes from blood samples could be used to detect phenotypic subgroups, which could provide a useful alternative to performing invasive bronchial biopsies to study pathophysiology in asthma patients. Mapper analysis of differentially expressed genes in asthmatic and control transcriptome profiles revealed several subtypes of severe asthma that differ in their response to treatment by corticosteroids.

##### 4.1.1.4. Traumatic brain injury.

A series of articles by Nielson et al. used Mapper to study the effects of traumatic damage to the central nervous system. Despite the fact that traumatic brain injury (TBI) and spinal cord injury (SCI) are major causes of disability and death, our ability to diagnose and treat these conditions remains extremely limited. In [107], Nielson et al. describe an application of Mapper to histopathological and clinical outcomes data that revealed novel interactions between SCI and TBI. Their analysis also identified perioperative hypertension as the most reliable clinical predictor of long-term recovery.

Subsequent work by the same authors [106] used a similar TDA-based method to identify subgroups of patients in a TBI database that includes imaging, genetic, and clinical outcome data. Their method successfully identified a subgroup of patients with mild TBI that are strongly associated with unfavorable outcomes several months after their initial injury. Analysis of the multivariate phenotype characterizing this subgroup revealed an increased prevalence of genetic markers related to cellular stress or DNA damage responses (PARP1) and striatal dopamine processing (aNKK1, coMT, DRD2) as well as increased rates of post-traumatic stress disorder in these patients.

##### 4.1.1.5. Oncology.

Granular patient subtyping is particularly useful in the domain of cancer diagnosis and management– it has long been established that heterogeneity at the genomic and cellular level gives rise to diverse subtypes of cancers that respond differently to treatment, so the use of precision medicine whenever possible is already standard practice in oncology. TDA has been used successfully in this domain to classify novel cancer subtypes, characterize tumor morphology, and predict prognosis in a variety of cancers. These applications, in particular of persistent homology, are described in a recent review by Bukkuri et. al. [22], so we will not repeat this discussion here.

We instead limit our overview to an early prototypical application of TDA by Nicolau et al. [105] for breast cancer subtyping. The authors propose an approach called progression analysis of disease (PAD) that combines Mapper and disease-specific genomic analysis (DSGA). DSGA provides a means of quantifying how much diseased tissue differs from healthy tissue, and has been shown to be extremely effective for analysis of genomic data. PAD uses DSGA to define the lens function of the Mapper construction, creating an analytic tool that clusters the data relative to the degree of deviation from healthy tissue. Applied to a high-throughput transcriptional microarray data set, their analysis identified a novel subtype of estrogen receptor-positive (ER+) tumors that had a statistically significantly distinct molecular signature from the rest of the data, but was not detected by clustering methods. The tumor subtype was characterized by high expression of *c-MYB* and low levels of innate inflammatory genes, and patients exhibit no metastasis and a 100% survival rate. In addition to detecting a novel *c-MYB* + breast cancer subtype, this study more generally demonstrates the utility of PAD on high-throughput biological data to potentially detect features that were missed by traditional analyses.

#### 4.1.2. Analysis of medical images

Image analysis was the subject of some of the earliest published applications of TDA dating back to the 1980s [78,40,136], and remains integral to many areas of research today. In particular, effective automated analysis could have interesting implications for diagnostic imaging, some of which are described below.

##### 4.1.2.1. CT & MRI.

Belchi et al. [11] introduced a method that uses persistent homology to describe the geometric structure of the system of airways in the lungs by extracting features from chest CT scans. The authors use demonstrate this tool in the context of chronic obstructive pulmonary disease (COPD), where they are able to classify COPD patients more finely than with current standard radiographic measurements. The TDA-based tool is able to differentiate between inspiratory and expiratory CT images, and its stratification of inspiratory scans agree with the standard GOLD guidelines for COPD classification. This level of stratification could not be obtained via other CT measurements such as emphysema score or airway diameter, supporting their conclusion that TDA methods can enhance standard diagnostic tools and inform more personalized treatments.

On a related note, a recent e-print by Iqbal et al. [73] documented the success of a TDA-based tool developed by the authors for automated detection of COVID-19 infection from CT images (see Fig. 8). Early detection has proven essential to minimizing the spread of COVID-19, so the ability to rapidly make an accurate diagnosis using CT would be preferable to slower laboratory tests such as RT-PCR. The method computes four different persistence diagrams for each image– three using VR complexes generating *H*_0_,*H*_1_, and *H*_2_, and one using a lower-star filtration. These complexes are able to capture important attributes of CT images including the shapes, textures and changes in intensity that characterize radiologic features of COVID-19 such as consolidations and ground-glass opacities. Their method achieved equivalent or better performance in terms of accuracy, precision, recall, specificity, and F1 score than current gold standard ML approaches such as deep neural networks. Further, this TDA tool circumvents the need for the large amounts of pre-classified data that are required to train most ML models.

In another application to medical imaging, Pedoia et al. [111,125] used Mapper to analyze MRI results in osteoarthritis (OA). MRI is a primary means of diagnosis in OA, but existing tools for assessing these images often fail to detect changes that occur in early disease stages. The method introduced here applied Mapper with PCA as the lens function to data including compositional MRI, MRI morphological grading, and biomechanics variables. In addition to a clear separation between OA patients and healthy subjects, Mapper illustrated different stages of OA as subnetworks, allowing for a characterization of OA phenotypes based on multiple variables simultaneously that would not be possible via traditional univariate analyses. As well as these multivariate patterns that may be useful for future research into pathogenesis and prevention, the TDA tool identified a novel predictor of cartilage lesion progression that could potentially serve as an imaging biomarker for early stages of OA.

##### 4.1.2.2. Histology.

A sequence of articles by Lawson et al. [83,82] develop a persistent homology-based system for automatic prediction of prostate cancer severity based on whole slide histology images. In [83], the authors compared their method to the current gold standard Gleason grading system for evaluating architectural subtypes in prostate cancer. They demonstrated that the TDA method is able to recapitulate the grades produced by the Gleason score while further identifying more granular architectural subtypes within a given Gleason grade. Subsequently [82], this method was used as a feature extraction tool in conjunction with ML algorithms. Using persistence images as topological summaries of histology in conjunction with a random forest classifier achieved relatively high performance on the task of classifying prostate cancer as high or low grade. Moreover, performance was only minimally affected by color deconvolution, indicating that this type of tool could be robust enough to be used with fewer such pre-processing steps.

Another series of articles by Rojas-Moreleda et al. [123,100] develop an analogous persistent homology tool for detecting segmentation of cell nuclei based on histology. Qaiser et al. [117] independently demonstrate the success of a very similar algorithm, lending further credibility to the approach. In a related vein, a novel index for immunohistochemical labeling based on persistent homology was shown by Takiyama et al. [139] to generate equivalent results to a trained pathologist for nuclear grading of invasive ductal carcinoma of the breast. Yet another study by Dunaeva et al. [52] combined TDA, geometric, and ML tools into a highly accurate method for classifying stomach lesions into oval, tubular, and irregular subtypes based on endoscopy images. Such applications demonstrate the potential of TDA for automated diagnosis and staging of cancers that eliminates the variability currently seen with inherently subjective visual analysis of histologic specimens by human pathologists.

#### 4.1.3. Improving diagnostic accuracy

Identifying correct medical diagnoses is often challenging, especially for complex patients with multiple comorbidities and diseases with ambiguous or variable symptoms. Since computational models can incorporate much more information than would be feasible to process manually, these tools could serve as valuable decision-support for challenging diagnoses. Several TDA-based tools for this purpose are described below.

##### 4.1.3.1. Pulmonary embolism.

Pulmonary embolism (PE) refers to a blockage of a blood vessel within the lungs by an object originating elsewhere, usually a blood clot that travels from a vein in the leg. This condition is not uncommon and has a mortality rate around 26% if left untreated. [7] Mortality can often be prevented with treatment, but delays in diagnosis are relatively common. This is due at least in part to the wide variety of clinical presentations and the non-specificity of the most common symptoms– shortness of breath, chest pain, hemoptysis, and elevated heart rate could all be indicative of any number of pathologies unrelated to PE. As such, the use of clinical prediction rules such as the Wells Criteria or revised Geneva score [104] is recommended for diagnosis. However, these types of criteria are somewhat subjective and difficult to standardize, and underdiagnosis of PE remains an issue. [76].

Motivated by this, Rucco et al. [126] developed an improved clinical prediction rule for PE based on TDA and artificial neural networks. Mapper was used to construct a topological model of 987 outpatients with suspected PE, as identified by documented risk scores (Wells or revised Geneva) in their clinical encounter records. Patients were classified as either healthy or having a PE based on angio-CT results. 26 clinical variables were used to build the Mapper, which showed two flares in the pathological portion of the network that corresponded to two distinct subtypes of PE patients. Mapper also identified a number of subgroups of healthy patients that likely correspond to non-PE diagnoses. Further, Mapper assisted in the identification of clinical variables that are most associated with a diagnosis of PE via feature selection by L-infinity centrality. These variables were used to define the input space for a back-propagation artificial neural network which was able to achieve higher accuracy than the gold standard risk scores commonly used in practice.

##### 4.1.3.2. Arrhythmia detection.

Improved cardiac monitoring and arrhythmia detection could significantly reduce time to diagnosis and improve outcomes for patients at risk for heart attack, which remains among the leading causes of death in the US. Dindin et al. [48] propose a topological approach to EKG monitoring using a multi-channel neural network that contains a TDA-based channel. Their use of persistent homology to reduce EKG signals to a simplified form has a number of advantages. First, it circumvents the need for complex and computationally expensive geometric feature engineering when training the neural network. Additionally, the stability properties of persistent homology lend a robustness to perturbations in EKG signals such as temporal expansion or contraction (e.g. due to bradycardia or tachycardia), which allows meaningful arrhythmic patterns to be detected regardless of these less meaningful differences. The authors benchmark the TDA-based neural network against gold standard arrhythmia detection methods, finding that their tool achieves equivalent or greater performance for both arrhythmia classification and detection of premature ventricular heartbeats.

### 4.2. Biology research

Notions of shape, connectivity, and continuity are intrinsic to biology. For instance, the ubiquitous paradigm that structure determines function relies on the idea that there is a strong relationship between the shape of a macromolecule or an organism and its behavior. Biology is also full of gradual, continuous changes such as progression from mild to severe disease or the transition of a stem cell from its pluripotent form to a mature differentiated state. Considering TDA is fundamentally based on the principle that shape and continuity within data have implications about the behavior of the underlying system, it is a natural perspective from which to study these types of phenomena, as is demonstrated by the examples in this section.

#### 4.2.1. Omics

##### 4.2.1.1. scRNA-seq.

Single-cell RNA sequencing (scRNA-seq) has revolutionized the field of transcriptomics by providing a mechanism to obtain RNA expression profiles for each cell in a sample individually rather than just the average expression across a population of cells. This better captures the heterogeneity of tissue samples and allows unique cell types to be characterized by clustering the individual expression data. However, data obtained from scRNA-seq are high-dimensional, high resolution, and often difficult to interpret with standard tools alone.

Clustering algorithms such as *t*-SNE are the most common approach to analyzing scRNA-seq data, but these methods are unable to capture the continuity that is intrinsic to the set of expression profiles– cells do not instantaneously flip from one discrete type or state to another, they instead gradually transition by incremental changes to gene expression and other processes. For example, suppose scRNA-seq is performed on a tissue sample taken from the border of a tumor. Clustering will identify one or more malignant cells types within the tumor as well as at least one normal cell type from outside the tumor. However, cells do not discretely jump from being normal to being malignant, the transition instead proceeds through a gradual accumulation of mutations until eventually the cell more closely resembles the malignant cells than the normal. A single-cell expression profile of the sample should therefore contain some cells in various stages of the transition, not just malignant and normal. Since clustering must place each point in a discrete cluster, it must choose between normal and malignant for each cell and thus has no way to reflect this continuous transition.

Mapper, on the other hand, conveys the same information about cell types as clustering, but also preserves the continuous structure indicative of cell type transitions and lineages. This was demonstrated in a study by Wang et. al. that used Mapper to analyze scRNA-seq data of melanoma tumor samples. [142] Fig. 9 illustrates the distinction between the output of Mapper and other clustering algorithms, where a numerical summary of the RNA expression profile was used a the lens function. Notice how Mapper depicts the malignant cell types (blue) as a branch off of a backbone of non-malignant cells (orange). This provides some additional insight into relationships and lineages between cell types, in particular which non-malignant cells are “closest” to being malignant and how changes in their RNA expression could move them toward or away from malignancy.

This potential of Mapper to provide insights into cellular development is also demonstrated in several other recent studies. Zechel et al. [147] used a PCA-based summary of scRNA-seq data as a Mapper lens to explore gene expression within the medial ganglionic eminence (MGE) of mouse brains. Using a method previously developed by the authors called single-cell tagged reverse transcription, expression data was spatially mapped back onto the images of MGE cryosections harvested from the mouse brains to create “transcriptome images”. This allowed assessment of the spatial arrangement of transcriptionally distinct subregions of the MGE, which likely correspond to functionally distinct areas within the tissue. The spatial patterns and functional predictions detected by Mapper in the transcriptome images were validated via *in situ* hybridization for a subset of highly expressed genes in each cluster followed by immunohistochemistry staining and functional assays, respectively.

An article by Setty et al. [134] introduces the Wishbone algorithm they developed for positioning cells along developmental trajectories based on multi-dimensional single-cell data, including scRNA-seq or mass cytometry. The authors demonstrate the ability of Wishbone to recover well-established stages of T cell development in the murine thymus based on 30-channel mass cytometry, then showcase the generalizability of the algorithm to other data sets including mass cytometry and scRNA-seq data from myeloid precursors in the human thymus. Compared to standard tools (SCUBA, Monocle, diffusion maps), Wishbone outperformed all other methods both in accuracy and resolution of precursor cell subtype ordering as well as identification of branch points along the cellular differentiation process.

Finally, an article related to murine embryonic stem cell (mESC) differentiation by Rizvi et al. [122] proposes single-cell TDA (scTDA) for unsupervised analysis of temporal, unbiased transcriptional regulation. The scTDA method outperformed all others tested, including the Wishbone algorithm described above, in its ability to correctly order precursor cell types along a simulation of a noisy, branched, asynchronous cellular differentiation process. The authors went on to study the differentiation of mESCs into neurons *in vitro*, showing that scTDA can be used to accurately recover and characterize the stages of this process. An established protocol was used to differentiate mESCs into motor neurons while extracting samples for scRNA-seq at several time points between pluripotency and maturity. On these data, scTDA was able to recover four distinct cell populations that arose during the differentiation process as well as the chronological ordering of these cellular states, again more accurately than any of the other aforementioned algorithms. Analysis of expression within these four subtypes was consistent with what is known about the underlying differentiation process, and the presence of markers identified by scTDA for each subtype was validated by immunohistochemistry. Further, scTDA was used to characterize specific transcriptional transitions occuring at each step of differentiation and the method was shown to be generalizable to additional data sets.

As a brief aside, supplementary analysis provided a biologically coherent explanation for the numerous loops present in the neural precursor region of the scTDA network– precursor cells differentiate extremely quickly upon induction by retinoic acid, meaning they are continually progressing through the mitotic cell cycle. Different proteins are needed to complete different parts of mitosis, meaning different genes are being expressed at each stage. This gives rise to periodic changes in RNA expression throughout the cell cycle, generating the loops observed in the precursor region. Neither pluripotent precursor cells nor mature motor neurons undergo this degree of mitosis, so fewer loops are present in these regions of the network.

##### 4.2.1.2. Global gene expression.

Most methods for analyzing global gene expression data involve clustering to identify subpopulations characterized by distinct expression profiles. However, such tools usually require large sample sizes and parameters that can be chosen arbitrarily, making their output unreliable. Jeitziner et al. [74] introduce an unbiased, TDA-based clustering method called Two-Tier Mapper (TTMap) that circumvents these issues. In particular, all parameters are optimized without requiring any input from the user that could introduce bias. Intuitively, their two-tier cover provides information at two scales– it uncovers the global clustering structure of the data (like Mapper), but also provides additional information about the composition of individual nodes, allowing TTMap to easily detect subgroups locally within a cluster as well as globally within the overall population. The utility of this feature was demonstrated by using TTMap to identify subgroups with differential expression of genes that vary along the human menstrual cycle. These genes are only expressed in certain phases of the cycle, corresponding to certain regions or nodes in the Mapper network, so these subgroups could not be detected using other tools. The authors go on to show that TTMap outperforms standard clustering in terms of both sensitivity and stability. Further, TTMap can be effective with much smaller sample sizes, and its output is robust to changes in input data and parameters.

##### 4.2.1.3. Multi-scale omics.

A recent paper by Zheng et al. [149] introduces HiDeF, a persistent homology-based method for studying the structure of biological data at multiple scales simultaneously. This work is motivated by the observation that data often has different patterns at different scales– scRNA-seq data, for instance, can be clustered coarsely to identify broad cell types or finely to identify narrower categories of cell subtypes. Most traditional approaches struggle to determine an optimal scale for analysis since it is generally impossible to predict at which scales patterns of interest can best be observed. This issue naturally lends itself to methods such as persistent homology that study data across a range of all possible scales.

HiDeF has three phases: (1) Represent data as a similarity network in which points represent individual biological entities and edges represent similarity between points. Then detect *network communities*, meaning densely connected subnetworks, across all possible scales. (2) Identify *persistent communities* that were identified at multiple scales. (3) Identify containment relationships where one community is fully contained in another. This results in a hierarchy of biological communities as output.

The authors first applied HiDeF to single-cell mouse transcriptome data, where it successfully identified known cell types as well as novel, more granular subtypes. Second, HiDeF was used to analyze protein–protein interaction networks, in particular to characterize interactions between viral subunits of SARS-COV2 and a subset of human host proteins. One of the persistent communities identified corresponds to a family of Transducin-Like Enhancer (TLE) proteins that interact with the Nsp13 RNA synthesis protein in SARS-COV2. This interaction could potentially form the basis for a drug target since TLEs are known to inhibit the WNT pathway, which has been shown to inhibit replication of coronaviruses. In addition to these examples, HiDeF could be similarly useful to study any system in which different structure emerges at different scales.

#### 4.2.2. Structural biology

Due to its intrinsic sensitivity to shape-based properties, TDA can be a valuable tool for studying the structure of biomolecules. TDA has been used to study RNA flexibility [116], chromatin structure and its response to damage [130,65,132,70], and biomolecular data analysis [97] in addition to the applications below.

##### 4.2.2.1. RNA folding.

Bowman et al. [18] use TDA to characterize intermediates in RNA hairpins, ubiquitous secondary structure motifs with many important biological functions. Though they are structurally quite simple, the mechanism by which they fold has been highly controversial. The authors use Mapper to provide insight into this question by identifying the distinct molecular states present in folding and unfolding pathways. They identify a number of intermediates that support experimental observations and recent literature indicates that the folding of these simple motifs is a complex, multi-state process.

##### 4.2.2.2. Protein structure & function.

TDA has also shown potential as a tool for exploring features of proteins including their 3D structure, flexibility [19], compressibility [59], and folding. An early application by Xia et al. [146] introduced the notion of *molecular topological fingerprints (MTFs)* based on the persistent homology of protein structural data. The authors demonstrate the utility of MTFs for protein identification or classification, quantitative analysis of flexibility and rigidity, and applications for characterizing the evolution of structural features throughout the protein folding process. This idea was extended in subsequent work using topological tools to classify whole proteins and individual domains by their structural signatures. [47].

Building on this foundation, inferring structure–function relationships of protein molecules based on their amino acid sequences or 3D geometric arrangement remains a prominent open challenge in biology. Motivated by this problem, Swenson et al. [138] propose PersGNN, a deep learning tool that incorporates aspects of graph representation learning and TDA. An advantage of this is its ability to capture both local and global structural features that would be missed using either method in isolation– on classification tasks predicting molecular function and gene ontology features from structural data, PersGNN outperforms pure TDA and graph representation learning techniques, as well as a standard neural network model.

##### 4.2.2.3. Protein dynamics.

Beyond static features of biomolecules, TDA has been used to examine dynamic processes such as protein folding. For instance, Kovacev-Nikolik et al. [77] used TDA to detect conformational changes between the open and closed forms of maltose-binding protein (MBP). *Persistence landscapes* [21] are a summary statistic for persistent homology developed to allow persistent homology to be used in conjunction with standard statistical analyses and ML kernel methods, both of which would be difficult or impossible using persistence barcodes or diagrams alone. These landscapes were constructed based on a coarse model of X-ray crystallography structures of MBP that represents each amino acid as a single unit. Subsequent analysis showed a statistically significant difference between the open and closed conformations of MBP that agrees with established physical models.

The authors go on to demonstrate how this approach can capture other dynamic properties of macromolecules such as ligand binding. In particular, the majority of residues involved in biologically important functions, such as those comprising active or allosteric binding sites, were shown to be located near the predominant loop in the persistent homology complex. These findings indicate the potential of such topological approaches to aid in the identification of key residues in other biomolecules based on their proximity to persistent topological features.

More recently, Ichinomiya et al. [72] developed a persistent homology-based method to analyze protein folding using complex data from molecular dynamics simulations. The method uses volume-optimal cycles to construct a *“topological feature vector” (TFV)* that is used as input for PCA and non-negative matrix factorization. In addition to producing results consistent with standard tools such as Cartesian co-ordinate and contact map analyses, the TFV approach successfully recapitulated the results of a previous study that demonstrated several distinct structural states for the protein chignolin (Fig. 10). Further, the volume-optimal cycles used here provide more information than persistence barcodes based on static structural data alone– their method was able to distinguish the native and misfolded states that were not distinguishable via barcodes generated by other analyses such as [146] discussed above. Moreover, outside this specific use case, the method is broadly applicable to studying the dynamics of other macromolecules.

#### 4.2.3. Immunology

The human immune system is extremely complex, comprised of a vast number of cell types and mediators that all interact to generate intricate responses to pathogens. In addition to being high-dimensional, these systems are full of nonlinear relationships between variables that present challenges for many analytic approaches. Further, although traditional differential equation models have been widely used across this field, such tools are susceptible to bias, errors in parameter fitting, and computational limitations, especially as models become more complex. Since TDA can examine data for patterns and features in an unsupervised manner (without the need for as many artificial assumptions that can cause bias), it can potentially provide an alternative approach that avoids some of these issues.

##### 4.2.3.1. Immune response to co-infections.

Recent work by Sasaki et al. [131] uses TDA to study immune responses during co-infections by multiple pathogens at the same time. Such responses are known to be extremely complex and potentially non-linear, making them difficult to study with standard tools. The authors used Mapper to visualize high-dimensional data pertaining to coinfection of influenza and pneumococcus in mice. They went on to identify persistent shapes within the resulting simplicial complexes for single viral infection, single bacterial infection, and co-infection. This indicated that the response of the immune system is indeed distinct for each of these infection scenarios, as depicted in Fig. 11.

Subsequent analysis revealed that the immune response to co-infection has three phases, the first corresponding to the viral infection, the second resembling a response to bacterial infection, and the third resembling neither of the single-infection responses. This work showcases how TDA can be used as a complement to standard modeling tools, especially for systems that may be too complex to model mechanistically without significant simplifying assumptions.

##### 4.2.3.2. Microbiomes.

The important role played by microbial communities, or *microbiomes*, in virtually every biological system has been a prominent topic of study in recent years. Applications of TDA to microbiome characterization date back to the mid 2010’s [115,4] and include work with implications for microbiome-based disease subtyping [49]. Since then, some success has been demonstrated using Mapper to model microbiome dynamics at various scales. One such study represents possible microbiome compositions as *quasipotential landscapes*, then uses these as input for Mapper. [29] Another uses Mapper to identify small-scale fluctuations in microbiome states that are relevant to large-scale changes in macroscopic phenotype, identifying state transitions that are associated with disease processes of the human gut. [30].

Finally, Liao et al. [88] introduce the *tmap* framework for studying microbiomes at the population scale. This type of analysis is challenging for traditional tools– both the space of microbiome features and the space of host characteristics are extremely high-dimensional, making it difficult and computationally intensive to attempt to identify patterns or significant variable associations. Standard methods either test each microbiome feature separately for statistically significant correlations with host phenotypes, which misses interactions between individual microbe features, or use dimension reduction and linear models to identify microbiome-associated host covariates, which may fail to capture nonlinear relationships within the data. Similarly, stratification of host phenotypes by clustering analysis also has significant limitations when applied to high-dimensional data that may contain nonlinear patterns. These issues motivate the use of topological tools such as *tmap* for this purpose.

The *tmap* algorithm first produces a compact Mapper representation of microbial variation from high-dimensional microbiome profile data. The values of a variable of interest are then mapped onto the Mapper network and used to generate numeric quantifications of subnetwork enrichment called SAFE scores. The SAFE score of a node for a given variable represents the enrichment level of the variable in the subnetwork centered around that node, meaning the SAFE scores across all nodes form a vector that quantifies local enrichment at each node in the Mapper network. In the final step, these vectors are compared for each feature and relationships between them are characterized. When benchmarked against other common microbiome analysis tools, *tmap* demonstrated the highest performance in detecting nonlinear microbiome associations in synthetic data, and successfully identified several nonlinear features that were not detected by the other tools.

Once validated, *tmap* was used to identify nonlinear and multiscale relationships in real-world data. For example, *tmap* was compared to traditional enterotype analysis for stratification of human gut microbiomes using data from the American Gut Project (AGP) (Fig. 12). Both methods were able to characterize global stratification patterns due to the variation in taxa comprising the microbial landscape. However, *tmap* also identified the more detailed local stratification patterns shown in Fig. 12a that more closely recapitulate continuous changes in the composition of microbiomes than discrete enterotype categories. Additionally, *tmap* avoids the requirement by enterotype analysis for the number of clusters to be specified in advance. Subsequent ordination analysis of SAFE scores revealed countries of host residence to be among the most significant covariates, and these were co-enriched with different microbial taxa (Fig. 12d). Finally, co-enrichements of other host factors and taxa appear to reflect lifestyle differences between residents of the countries (Fig. 12e,f). This analysis provides an example of how *tmap* can be used both to stratify microbiome profiles and to characterize relationships between host factors that contribute to this stratification.

#### 4.2.4. Neuroscience

Alongside disease subtyping and image analysis, neuroscience is one of the biomedical fields featuring the most published TDA research. Applications range from brain mapping [128,84,12] and neuroimaging [113,24,61,143,15] to identifying [124,91], modeling [34,62,109,81], and predicting outcomes [43,57] in disease phenotypes. Though many of these are unique and interesting, we refer to other reviews [44,137,35] for a thorough discussion.

### 4.3. Public health & healthcare optimization

In addition to its applications to precision medicine, TDA can be used in the domain of public health for population-level analyses that remain sensitive to variation between subpopulations.

#### 4.3.1. Epidemiology

Epidemiological models present a number of challenges– systems are often highly nonlinear, models must adapt to new information being gathered in real time, and the scale of the data is generally very large. Tools from statistics, dynamical systems, network analysis, and ML were designed to account for such issues, but here we describe several recent studies that examine the use of TDA as a complement to these approaches.

##### 4.3.1.1. Contagion modeling.

Taylor et al. [141] introduce a method based on TDA and nonlinear dimension reduction to construct “contagion maps” to study the spread of processes across a network. The authors use the Watts threshold model (WTM), a well-established dynamical systems model for contagions, to generate filtrations of networks that can be used for persistent homology computations. This framework provides an approach to modeling spreading processes that allows for analysis of the geometry and topology of contagion networks simultaneously.

A few other publications document the use of TDA to examine the epidemiology of specific diseases with varying degrees of success. These include studies of influenza [42] and Zika [89] viruses via persistent homology as well as a recent preprint that describes the use of Mapper to model the spread of COVID-19.

#### 4.3.2. Healthcare optimization

As has been illustrated in the ongoing COVID-19 pandemic, determining allocation of medical resources during times of high demand presents a nontrivial challenge for the healthcare system. The development of computational tools such as the one described below could prove highly beneficial for optimizing this process to coordinate the best possible response of medical organizations to future public health concerns.

##### 4.3.2.1. Patient flow analysis.

Dugast et al. [51] recently used TDA as the basis for a real-time computational method to estimate the number of abnormal arrivals likely to present to the emergency department (ED). Such abnormal arrivals tend to occur during public health events such as disease outbreaks or epidemics, and this sudden increase in patient flow can lead to overcrowding and resource shortages in hospitals. Their proposed method seeks to identify moments in time where ED arrivals are likely to increase as early as possible, giving healthcare organizations more time to prepare for the increased flow. The authors used detrended fluctuation analysis (DFA) followed by persistent homology to detect and characterize variability in ED admissions over time. Their TDA method outperformed standard algorithms for change detection (CUSUM, control charts methods) by detecting the increase resulting from an epidemic event at the same time as or earlier than other tools, but with a lower rate of false detection stemming from high variation in the data.

## 5. Strengths & weaknesses

Armed with a basic understanding of the inner workings of TDA algorithms and some of their applications, we can develop a better appreciation for their benefits and drawbacks as well as the types of problems that may warrant a topological approach.

### 5.1. Advantages of topological tools

TDA views data from a fundamentally different perspective than other tools by focusing on topological features and patterns. Intrinsic to this unique perspective are properties that confer certain advantages in the context of big data and biomedicine.

#### 5.1.1. Novel feature discovery

TDA may recover features from data that were overlooked by other methods, in particular shape-based properties that would be more visible from a topological vantage point than a geometric or statistical one. For example, the data sets shown in Fig. 1 were identical in terms of descriptive statistics but are easily distinguishable by TDA since they differ in homology. This was also illustrated by several real-world examples from Section 4, such as those where TDA was used to identify novel disease subtypes. [87,107,105] Further, TDA is able to capture higher-dimensional structure than most other methods. This proved important for applications to fields such as immunology where high-dimensional, nonlinear relationships abound and have significant implications for the behavior of the underlying biological system.

#### 5.1.2. Multiscale models

A key strength of TDA is its ability to identify and characterize structures at multiple scales simultaneously. This is particularly natural for persistent homology, which is fundamentally based on an assessment of topological features across a range of parameter values. The utility of this is illustrated by the applications that used persistent homology to build multiscale models such as the HiDeF method for studying omics data at various scales [149].

For Mapper, it makes sense to think of the algorithm as a type of “enhanced clustering”– like traditional clustering, Mapper detects subgroups of points with similar attributes. These subgroups represent connected components, or 0-dimensional topological features. However, unlike standard clustering, Mapper also illustrates higher-dimensional information about how these subgroups inter-relate and fit together to form the overall population. In this way, Mapper is able to integrate local information (subgroups of similar data) into a global model (their relationships within the full data set). This is clearly demonstrated by the *tmap* algorithm [88] or in the scRNA-seq applications described in Section 4.2.1, in which Mapper is used to visualize the evolutionary process from one cell type into another.

#### 5.1.3. Reduction of size & dimension

Topological tools are one possible approach to the challenges surrounding dimension reduction. TDA has proven to be more computationally efficient and effective for this purpose in some respects, identifying hidden structures within data that were not discovered by standard methods. [90] Both persistent homology and Mapper convert large, high-dimensional data into smaller, lower-dimensional representations that are much easier to manage. In contrast to traditional methods, which are not always guaranteed to maintain meaningful features after dimension reduction, this compressed representation encodes all important topological information from the input data in a compact, simplified form.

#### 5.1.4. Coordinate & deformation invariance

Topology considers shapes in a *coordinate-free* manner, meaning topological properties do not depend on any choice of coordinate system. Similarly, topological properties are unaffected by continuous deformations such as scaling or translation. In the context of biomedical research, this confers an ability to circumvent certain issues related to noise and reproducibility– if the same information about the object is collected by two different devices or experiments, its representation may differ slightly even if the data describe the same object. For instance, one X-ray crystal structure of a protein may be rotated, translated, or viewed from a different angle as another. These types of changes could cause issues for geometric methods that depend heavily on coordinate-based representations, but this effect would be diminished for TDA tools. Another benefit of this invariance is illustrated in the application using TDA for arrhythmia detection [48], where persistent homology was able to detect features in EKGs independently of most meaningless signal perturbations that would cause problems for other tools.

#### 5.1.5. Symbiosis with ML

Models that use TDA in conjunction with ML are able to harness the impressive accuracy of ML tools while retaining many of the other strengths of TDA, often leading to improvements in performance and interpretability. First, it has been shown that augmenting ML models with TDA components can improve predictive accuracy over models built on either approach in isolation. This augmentation can be done in several different ways. One option is to add a topology layer to an ML model such as a neural network, which has been shown to increase model accuracy [20]. TDA can also be used to refine neural networks after training to boost their performance [102]. Another approach [58] uses Mapper to examine the topology of convolutional neural networks, finding that augmenting an untrained network with information about the topological structure of a trained network decreases training time while increasing accuracy and generalizability to new data.

TDA can also be used to improve explainability and interpretability of opaque ML models such as in [6]. Using the probabilities generated by a ML classifier as the lens for a Mapper construction, the resulting simplicial complex is a visual representation of how the model classifies data overlaid onto a network depicting similarity between data points (Fig. 13). This provides a degree of insight as to how the ML model makes decisions– areas of the complex comprised of a single category of data represent classes that are easily identified and distinguished, whereas areas containing a mixture of categories represent data classes that are somehow ambiguous or difficult for the model to tease apart. Moreover, paths from a node of one class to another of a different class illustrate how changes in baseline features affect its position in the network and therefore how it is classified by the model.

On the other hand, ML tools can be useful components of TDA pipelines. For instance, it is relatively straightforward to define a notion of distance between persistence diagrams, allowing them to be used in conjunction with ML algorithms. Since a persistence barcode or diagram is essentially a topological signature for a data set, ML models can be trained to distinguish between data sets with different persistent homology. There are a number of ways to convert the output from persistent homology tools into appropriate vectorized input for machine learning. The details of these methods are ommitted here, but they include persistent landscapes [21], persistence images [2], persistent curves [38], and kernel functions [41]. Several open-source software toolkits such as *giotto-tda* [140] have also recently been developed to integrate TDA into ML models.

#### 5.1.6. Data exploration

TDA can also be a valuable tool for exploring new data sets. Mapper has proved especially well-suited for this, both alone and in conjunction with ML models as described above. Part of this is due to its aforementioned ability to integrate local information into global population models, producing visualizations that incorporate both perspectives simultaneously. Another part is that the simple topological summary produced by Mapper provides a mechanism to explore data that are far too complex and high-dimension to easily visualize through graphs, plots, or other similar methods. Further, these summaries can be viewed with respect to any property of the data, allowing for visualization of how that property affects the position of a data point in the overall population. This can be very useful for qualitative assessments as well as for hypothesis generation, as is illustrated by applications including disease subtyping [27,107] or examining immune responses [88].

#### 5.1.7. Stability

Finally, the theoretical framework upon which TDA algorithms are based allows for precise quantification of their *stability*, meaning how their output is affected by changes to input data and parameters. Intuitively, a method is *stable* if small changes in input result in analogously small changes in output. In other words, stable methods should produce similar outputs for similar input data and parameter values. Stability properties also confer a measure of robustness to certain issues of data quality since the impact of any noise is guaranteed to be limited. A substantial body of literature describes and quantifies the stability of TDA methods in many practical contexts [39,26,10], but this challenge remains is an active area of research. The related issue of sparseness can be highly problematic– missing information in even a fraction of variables for a fraction of data points can create significant artificial voids when viewed in high dimensions, making it extremely difficult to recover features from high-dimensional data without sufficient coverage of all variables. Though such artificial areas of sparseness may be detected as features, many will not persist over many parameter values and can thus be identified as noise. Even in situations where artificial voids are as persistent as true features, different data sets can still be distinguished by their unique persistent homology signatures. TDA can therefore still be useful even when data is too sparse to allow for full recovery of all features.

### 5.2. Limitations of topological tools

#### 5.2.1. Validation & accessibility

TDA still has a long way to go in terms of validation, widespread use, and accessibility. One contributing factor is the general lack of awareness about TDA in disciplines outside of mathematics, possibly due to its somewhat complex and unapproachable theoretical underpinnings. As a consequence, there are relatively few published applications to research in the life sciences and other fields. Then since there is minimal domain-specific literature to reference, it can be difficult to find accessible explanations about what TDA is or how it can be used. Widespread use is also limited by the fact that it can be quite challenging to use and interpret the results of TDA tools in practice. Many of the published studies that do exist use closed-source software that is inaccessible to most researchers, but using open-source tools requires significantly more technical know-how. In addition to processing data into valid input and adapting algorithm components to suit domain-specific needs, researchers must learn how to interpret the physical or biological relevance of unfamiliar topological results. A related side-effect is a lack of validation and benchmarking of TDA against standard tools in most scientific fields, meaning it can be difficult to predict how successful TDA might be on new research problems in these areas.

#### 5.2.2. Parameter tuning

A related issue is that of parameter tuning, especially for less conventional applications of TDA such as biomedicine. Both Mapper and persistent homology require several choices for parameters to be made by the user. These choices can dramatically affect output, but given the lack of published precedent and computational expense of rigorously tuning multiple parameters, it can be difficult to find optimal values. Until more literature on domain-specific TDA emerges, some experimentation may be required to choose values that effectively capture features of interest.

One key component required by both algorithms is a metric on the input data, meaning a measurement of distance or similarity between data points. In mathematical settings, a natural choice for this metric is often clear. For example, the standard notion of distance between points *x* and *y* in a 2D plane is given by $\sqrt{x^{2}\;+\;y^{2}}$. On biological data, however, it is not always clear how to formulate a metric that faithfully reflects whatever notion of similarity one hopes to capture– an appropriate concept of distance between patients represented as vectors of clinical variables is less intuitive than for points in the plane. The problem of defining metrics on non-traditional data types arises in many areas outside of TDA and is addressed by a growing body of literature, but this area could benefit from additional work going forward.

Mapper also has a number of other user-specified elements such as the lens function, cover, and clustering method. Further, each of these components may require its own parameter choices such as a desired number of cover sets or a clustering cutoff threshold. If any of these elements is poorly-suited to the underlying data, the output may not be terribly illustrative. For instance, in the example from Fig. 4, if the cover was chosen to have only two overlapping sets, the resulting simplicial complex could have only two nodes connected by an edge, which would fail to capture the loop feature of the input data.

#### 5.2.3. Robustness

Though TDA is relatively robust to noise, it is not immune to this challenge. Stability properties guarantee that any changes in output are bounded relative to perturbations in input. However, these perturbations can be very large, especially on high-dimensional data– if each of the *n* coordinates of a data point is perturbed by some small value ∊, in *n*-dimensional space the point actually moves distance ∊^*n*^ away from its original position. This change by ∊^*n*^ is much larger than the change by ∊ in any given coordinate, meaning small perturbations to individual variables can end up having a much larger effect in higher dimensions. Dimensionality reduction (e.g. PCA) is commonly performed before TDA to mitigate this issue, but it can not eliminate noise entirely and some information may be lost during the process. Additionally, Mapper in particular may only provide meaningful output when parameters are within a certain range, and it is not always clear how to choose optimal values that fall within that range. Such choices also require a deep understanding of the inner workings of the algorithm, which may be unreasonable to expect from less technical users.

#### 5.2.4. Computational complexity

TDA can also be quite computationally intensive. Any persistent homology algorithm must calculate a great many simplicial complexes corresponding to a range of parameter values, and the number of nodes in these complexes increases steeply with data size and feature dimension. Novel software [93,9,148] and methods [152,98,145,16] have been developed in an effort to simplify these computations in some situations for certain types of data, but this remains an area of ongoing research. As such, it is common to limit calculations to only the first few Betti numbers.

Mapper is generally quite efficient relative to other tools, but some components may cause computational limitations. Even though part of the purpose of the lens is to reduce dimensionality before intensive computations occur, calculating covers can still require significant processing power, especially for higher-dimensional lens spaces. The clustering step is another source of issues as it requires calculating pairwise distances between large numbers of data points. Although Mapper avoids performing these calculations on the entire data set by clustering only within pullback sets, these sets can still be quite large. Less demanding components can often be substituted if issues occur, but computational challenges can never be completely eliminated when working with big data.

#### 5.2.5. Loss of geometric information

The fact that TDA is insensitive to variation in many geometric properties can be both a blessing and a curse– on one hand, it confers useful features such as coordinate invariance and robustness to noise. On the other hand, topological methods may be unable to detect such properties when they *are* relevant. This is often not a huge issue in practice, however, since TDA can be used in conjunction with other techniques when this type of information is required, for example by using a geometrically-sensitive Mapper lens. [136].

## 6. Discussion

### 6.1. Summary: When to Use TDA

TDA is most useful to study systems that cater to the advantages of a topological perspective, such as those with continuous or shape-based structure that emerges at multiple scales. TDA tools are uniquely able to concisely summarize this structure in a way that often detects features missed by other methods. TDA can also be used as a dimensionality reduction tool that preserves essential topological relationships, or in conjunction with ML models to improve their accuracy and ability to generalize to new data. Additionally, TDA may be useful on certain types of data that present challenges for standard tools since it more robust to certain data quality issues such sparseness and noise, especially on high-dimensional data. Further, TDA can be used on smaller data sets than would be sufficient to train ML models or produce adequate power for statistics.

More specifically, Mapper is particularly useful for exploratory analysis and hypothesis generation, especially in situations where the data or underlying process have some type of continuous structure such as with disease progression and developmental or evolutionary processes. Persistent homology, on the other hand, is a natural framework for quantifying the topological structure of data and examining this structure at many different scales simultaneously. It is especially well-suited for multiscale modeling or for building unique topological fingerprints of data sets that can be used to distinguish them from each other. Fig. 14 and Table 2 summarize these methods and their applications in a biomedical setting.

### 6.2. Goals for the future of TDA in biomedicine

As we have seen, TDA can be a powerful tool across a diversity of applications; but despite its successes, development and utilization of TDA in the life sciences has been relatively limited. There is still a great deal of important work to be done in simply extending established applications such as those in Section 4 to new data or closely related problems. Outside this context, we will discuss a few examples of underdeveloped research areas that could potentially benefit from incorporating TDA.

#### 6.2.1. Improving TDA tools

##### 6.2.1.1. Validation & benchmarking.

As established above, there is a pressing need for published comparisons of TDA to other tools in the biomedical sciences– researchers would be more likely to adopt a new tool, especially a theoretically unapproachable one, once it has been validated and its performance benchmarked against standard methodologies in the field. This will require computational research to verify that TDA can recover known results with equivalent or better performance than standard methods, but also experimental studies assessing the biological plausibility and interpretations of results obtained from TDA.

##### 6.2.1.2. Domain-specific development.

Most algorithms rely on a few components and parameters that must be chosen by the user, which can be beneficial since it allows domain-specific knowledge to be incorporated into the construction. However, research about how to develop adapted components, choose parameters, or optimize TDA algorithms for specific applications is in short supply. This presents a significant need for future work establishing TDA approaches tailored to more diverse research problems, especially outside the field of mathematics.

For instance, both Mapper and persistent homology rely on a *metric*, meaning a function specifying how to measure the distance between points in a data set. This is straightforward for data that already comes equipped with a natural notion of distance such as points in standard *n*-dimensional space. Unfortunately, defining an appropriate notion of distance or dissimilarity is less trivial in biomedical contexts where points represent cellular gene expression profiles or patient EHRs. A small body of research begins to address this issue in select fields [110,150,35], but significantly more work is needed in this area. Development of other elements such as lens functions and covering methods for Mapper that are sensitive to issues related to domain-specific data would also be beneficial.

##### 6.2.1.3. Usability.

A major barrier to widespread adoption of TDA is the lack of publicly available software tools with both useful functionality and an approachable interface for users without a strong technical background. Many published applications make use of proprietary TDA software developed by Ayasdi that may not be accessible to all researchers and data scientists. There are several public, open-source implementations of Mapper [114,101,1] and persistent homology [108,1] algorithms. However, none are particularly usable without the ability to implement appropriate data pre-processing, run code from the command line and perform basic debugging. Additionally, using or modifying these tools requires a reasonably advanced understanding of each step of the algorithm its associated parameters. Development of TDA software that is functional but still straightforward to use would therefore be a huge step toward broadening the use of TDA outside of quantitative fields.

#### 6.2.2. Analysis of time series data

Persistent homology is a natural framework for analysis of data that changes over time since it is intrinsically equipped with notions of birth and death as features appear and disappear. The use of TDA on time series data is a relatively underdeveloped area of research that could benefit from further growth– a few recent studies [120,129,36,112,121] make use of persistent homology for this purpose, but hardly any do so in a biomedical context [37].

#### 6.2.3. Explainable machine learning

ML has already proven to be extremely useful in biomedicine, surpassing human performance on tasks such as clinical diagnostics and outcome prediction [75,99,33,92,118]. However, as we have seen, these models have important drawbacks related to interpretability. Translating and extending the explainable ML framework in [6] could provide a mechanism to harness the power of existing, highly accurate ML tools to create interpretable, visual models that can be easily explored.

For example, Fig. 15 depicts what it might look like if an ML tool trained to predict mortality risk from EHR data was used to construct a Mapper lens. Nodes would be comprised of phenotypically similar patients and colored by level of risk, making it simple to identify and characterize subtypes of high risk patients or explore differences in phenotype between low risk and high risk areas of the network. Examining how clinical variables change along paths from low risk to high risk regions could provide insight into disease progression or pathophysiology, while examining paths from high risk to low risk areas could inform hypotheses about which variables would need to be modified by a potential treatment to decrease a patient’s risk. This would be particularly useful in settings such as critical care where patients tend to be immensely complex with many risk factors and comorbidities, but clinical interventions can have a significant impact. Extending this example, different ML tools trained to predict other outcomes could be used similarly– for instance, a lens based on a tool that classifies histology images into tumor subtypes could be used to explore the gradual histologic changes that occur as the cancer progresses or to assess which tumor subtypes are most similar or distinct.

#### 6.2.4. Digital twins

One possible culmination of the personalized medicine idea is the *digital twin*, meaning a computational model that precisely recapitulates an individual’s unique physiology. If perfected, such a model could generate tailored predictions about clinical outcomes or disease course that account for the full complexity of a given patient, and diagnostic tools or treatment regimens could be experimentally tested *in silico* without risk or harm.

Of course, even the most cutting edge tools that exist today are still a long way off from this. Such a model must integrate large, heterogeneous, multi-scale, real-time data about a patient’s physiology and disease progression. Since TDA has already proven successful at handling many of these challenges individually, it may eventually play an important role as technology is begins to emerge in the coming years.

### 6.3. Guide for getting started with TDA

Finally, we would like to highlight a few resources that may be helpful to readers who want to learn more about TDA or how it might be applied to their own work.

Before delving into practical considerations such as software, potential users should ideally understand the methods well enough to form an idea of how they hope to use TDA. A few foundational articles introduce the topic of TDA in general [25] and Mapper [136] or persistent homology [63,108,96] in particular. Though somewhat long and technical, these provide a good comprehensive reference that can supplement other resources.

For an even more in-depth initiation, the book “Topological Data Analysis for Genomics and Evolution” [119] provides an excellent introduction to the basic mathematical ideas used for TDA as well as a thorough discussion of many biological applications. Though detailed and at times technical, we feel the majority of the book is reasonably accessible to both mathematical and non-mathematical audiences. Alternatively, the books “Computational Topology” [55] and “Elementary Applied Topology” [64] may be more difficult for non-technical readers to digest, but are freely available online. Other resources including tutorials, blogs [133], and YouTube videos [5] may provide more approachable (though not peer-reviewed) learning options that can serve as a primer for some of the more rigorous references.

After understanding the basics of the algorithms, we hope that reviews such as this one and others [31,144,22,44,137,35] can help readers identify plausible applications of TDA in their field of research. Determining specific workflows for data pre-processing and parameters tuning may still be challenging, especially for atypical biomedical data, so it may be useful for new users to initially experiment on simple synthetic data sets to get a practical sense for how TDA can be implemented before moving on to working with complex data types.

As for performing the actual analysis, there are many publicly available software packages that can be used for TDA. We do not attempt to describe them all here, but more comprehensive lists of TDA software packages can be found online [1,66,3]. Though most freely accessible TDA tools do require some amount of technical ability, the GUDHI library [93] might be a good place to start for users interested in persistent homology. GUDHI comes equipped with a large set of data structures and algorithms designed for TDA, and the library includes several data sets and detailed examples that are helpful for understanding practical TDA workflows. GUDHI’s source code is also well-documented with a Python interface that is relatively easy to install and straightforward to use.

For those interested in getting started with Mapper, Mapper Interactive [151] is a very usable tool that allows users to modify input and parameters and view the resulting output via a simple, web-based interface. Though somewhat less flexible than the lower-level software libraries, this type of tool provides a great way to easily explore and visualize how changes to data and parameters affect the output of Mapper without needing to do much programming. Finally, for users who are familiar with machine learning, *giotto-tda* [140] provides a set of tools targeted at using TDA in conjunction with ML.

## 7. Conclusion

As we continue to move toward more data-driven biomedicine, TDA can be a valuable tool for everything from straightforward applications in image analysis or structural biology to more ambitious goals like building personalized digital twin models. Its versatility and ability to integrate heterogeneous information across multiple scales confer a unique perspective that will render TDA an essential tool as computational models of the future begin to catch up to the recent big data explosion.

## Figures and Tables

**Fig. 1.**
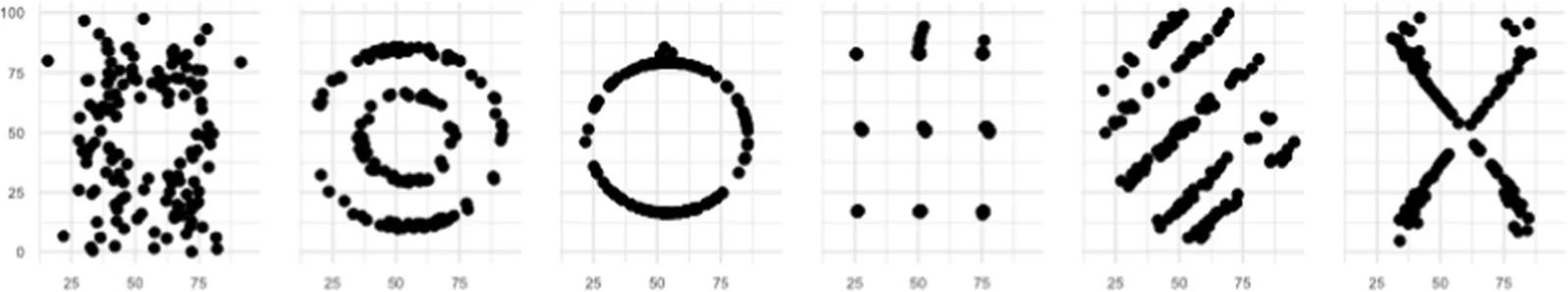
All six of these data sets have identical summary statistics (*x/y* mean, *x/y* standard deviation, and Pearson’s correlation) to two decimal places, but are clearly visually distinct. All data was taken from [95].

**Fig. 2.**
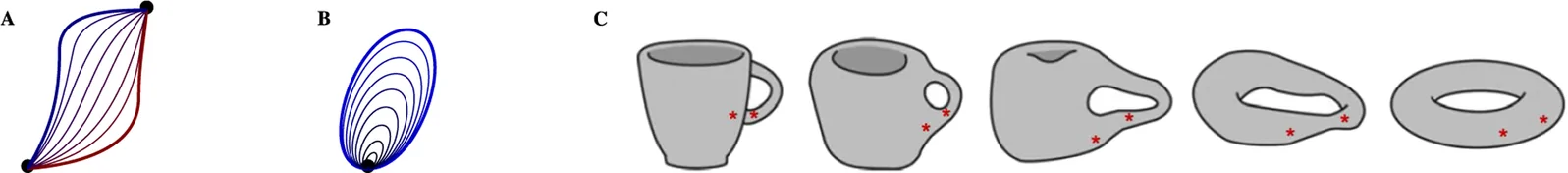
A few classical examples of topological equivalence by continuous deformation. **A.** The red loop can be stretched and bent to form a curve identical to the blue loop, so these loops are topologically the same, despite being visually and geometrically different with different sizes and curvatures. **B.** The loop can be contracted down to a point. **C.** The transformation depicted here is the basis for the famous joke that to a topologist, a coffee cup and a donut are identical– the shape on the left and the shape are the right are indeed topologically equivalent since one can be transformed into the other through only bending and stretching.

**Fig. 3.**
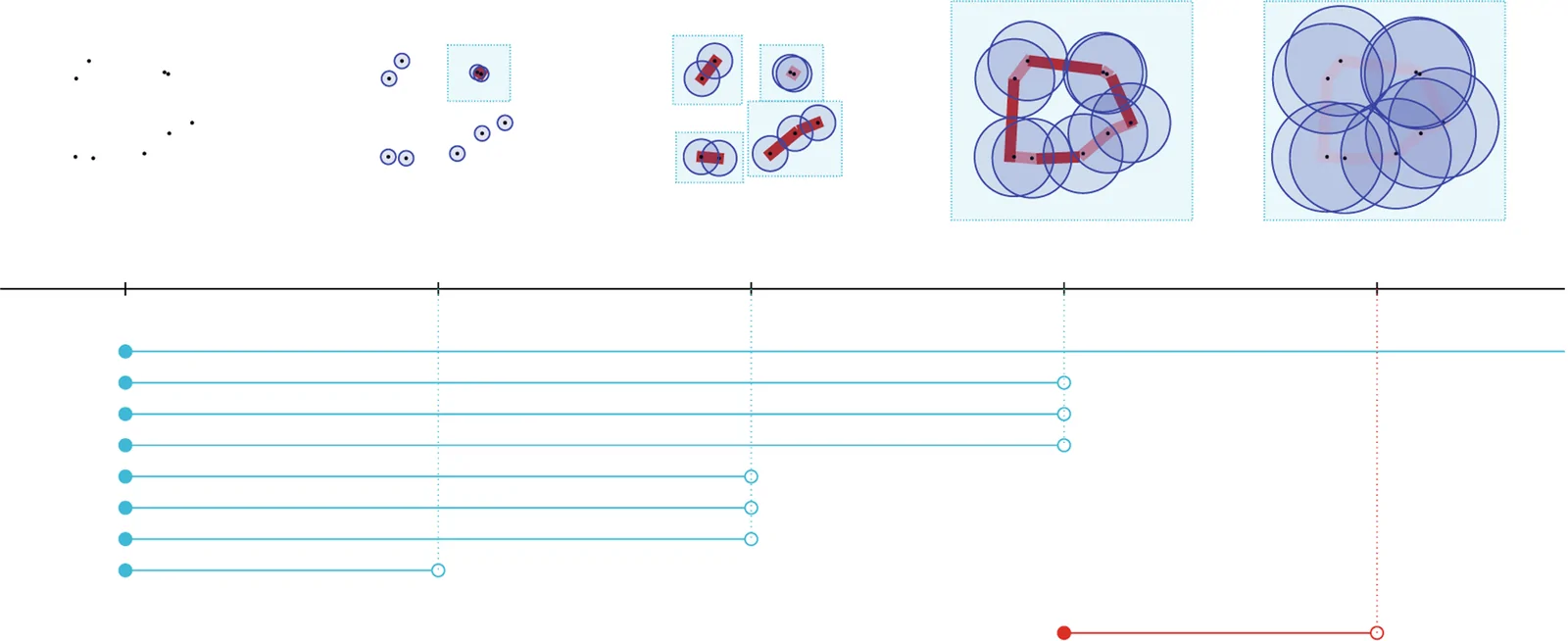
Visualization of persistent homology. The input data set *X* is shown on the far left. In this image, each of the 8 points in *X* is its own separate connected component, producing the eight 0-dimensional features depicted in the barcode below. In the next image, balls of some small radius are drawn around each point. A line is drawn between the two points whose balls overlap in the top right of the image, combining these two points into a single connected component, causing the death of one 0-dimensional feature depicted as the open circle below that image. As the radii of the balls increase, more components combine until only a single remains. The third image also depicts the birth of the only 1-dimensional feature, when the lines between points connect to form a loop. The death of this feature occurs when the radii are large enough that this hole is filled in.

**Fig. 4.**
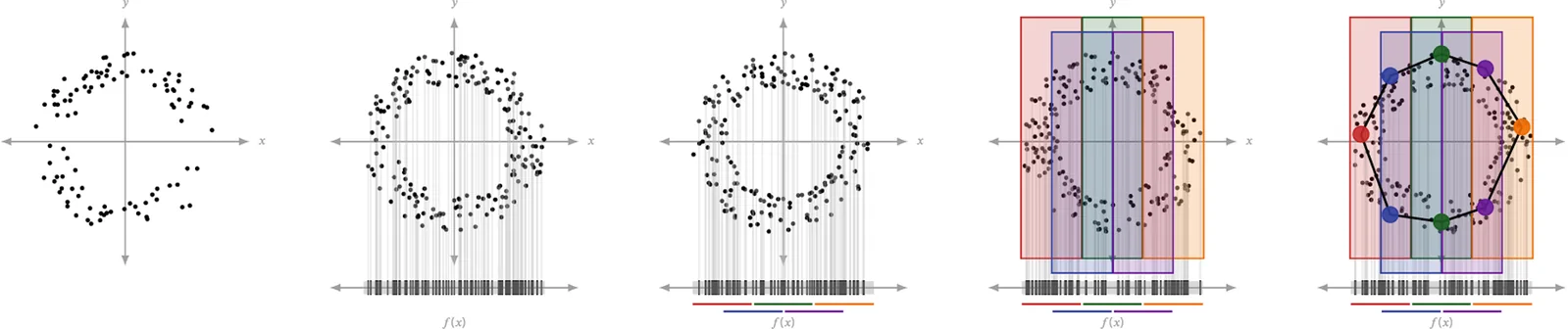
Visualization of the Mapper algorithm. **A.** Input data set *X* is a point cloud roughly distributed around a circle, **B.** Applying filter function *f* : *X*⟶*Z* = _R_ maps each point in *X* to its *x*-coordinate. These lensed values are displayed as vertical lines in the space below *X*, **C.** The image *f*(*X*) in lensed space is covered by overlapping intervals, each of which is shown in a different color, **D.** The colored boxed depict the pullback of *f*(*X*). Each box surrounds only the points whose lensed value is within the cover set of the same color. **E.** Clustering points *within each pullback set* and representing each cluster as a node (0-simplex) gives the points of the simplicial complex. If two clusters have any points in common, an edge is drawn between their corresponding nodes.

**Fig. 5.**
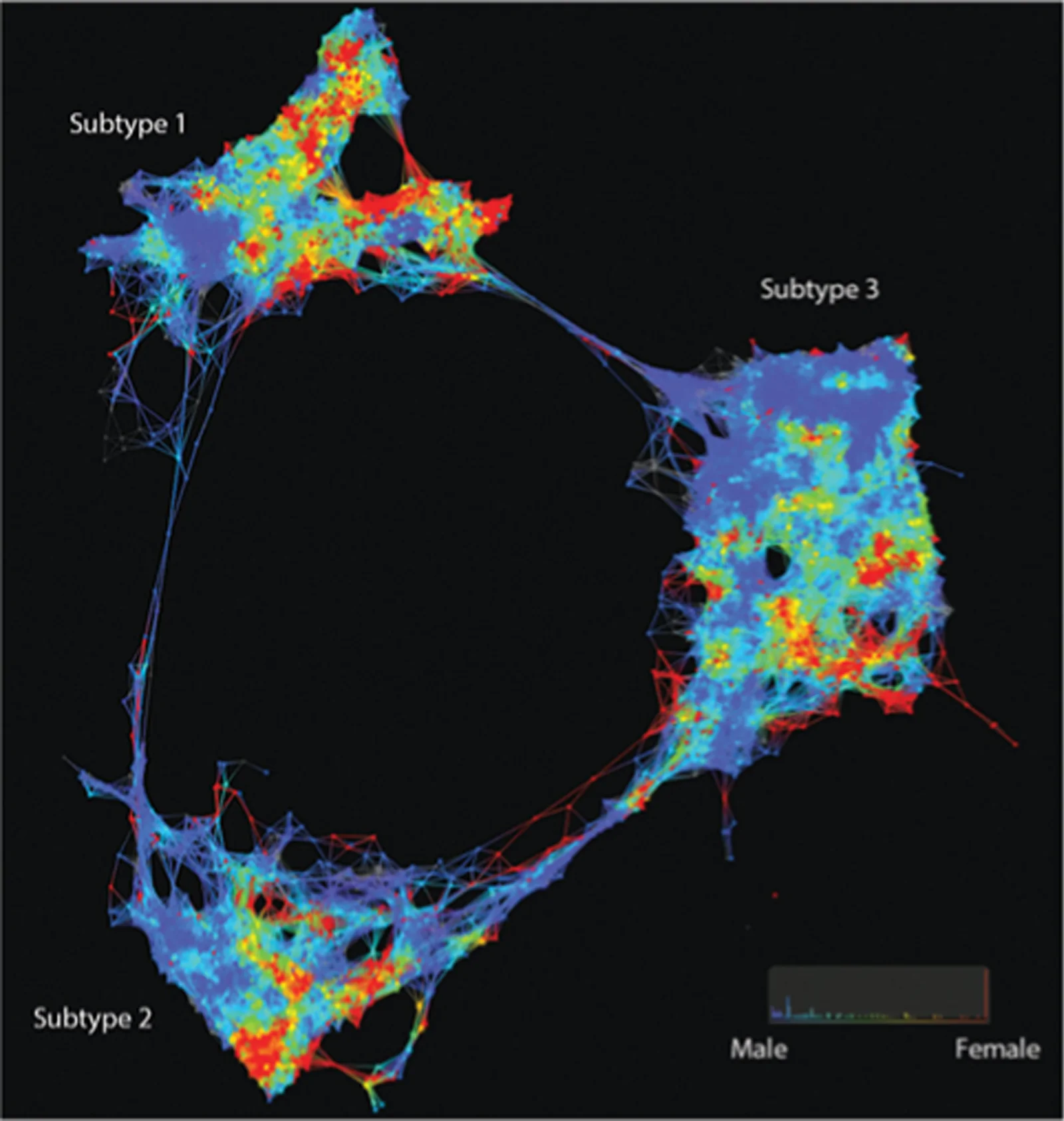
Patient-patient network for topology patterns on 2551 T2DM patients. Each node represents a single or a group of patients with significant similarity based on their clinical features. Nodes connected with edges indicate the nodes have shared patients. Red color represents enrichment with females, blue enrichment for males. Adapted with permission from [87].

**Fig. 6.**
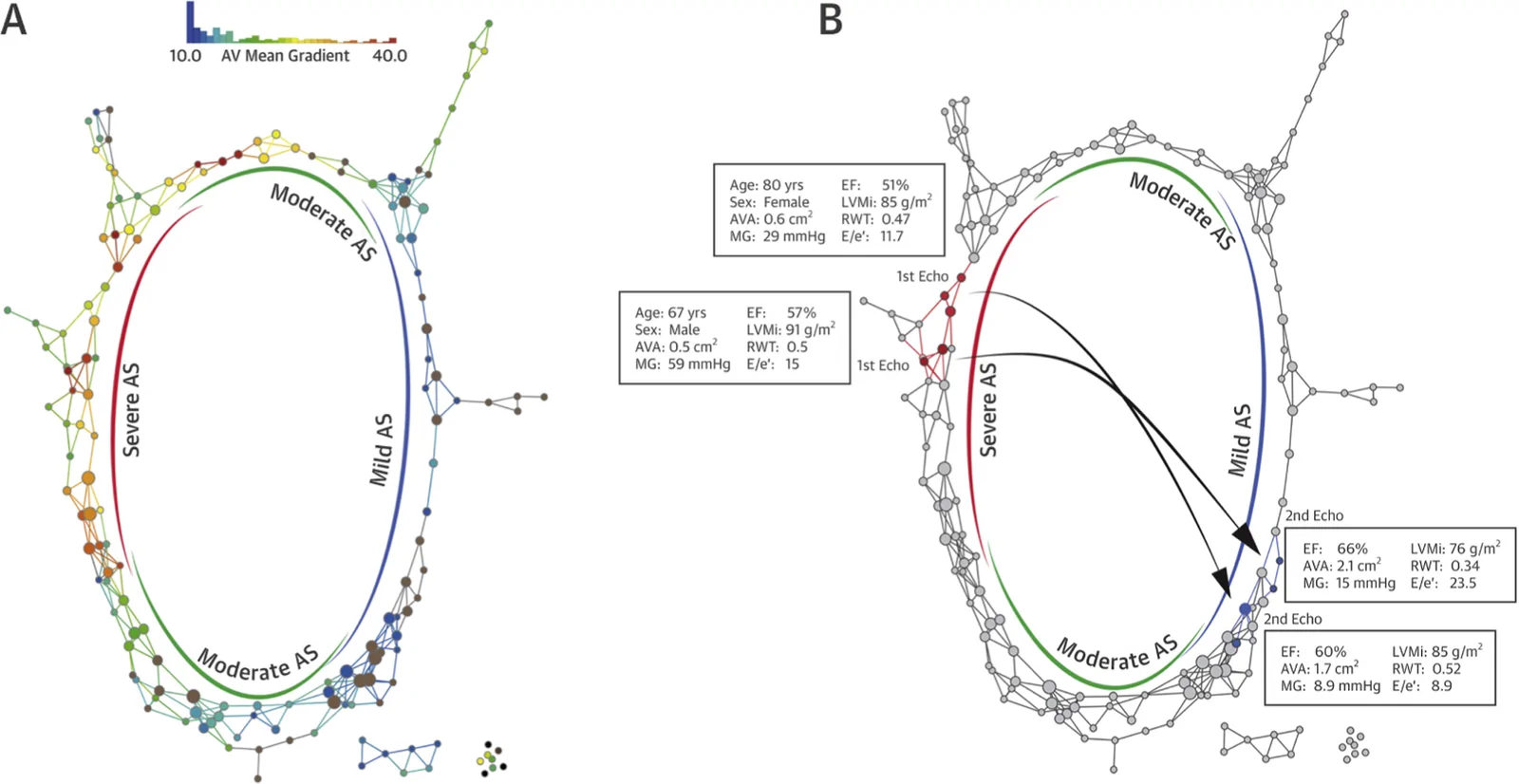
Patients in Network After Aortic Valve Replacement (AVR). **A.** Nodes containing patients imaged after AVR (brown nodes, median follow-up days: 36; range: 15 to 62 days) are depicted over the network and seen to aggregate in the region of mild or moderate AS. **B.** Inter-region motion of 2 representative patients (64-year-old man and 80-year-old woman). AVA = aortic valve area; Ao. = aortic; EF = ejection fraction; LV = left ventricular; RWT = relative wall thickness; LVMi = left ventricular mass index; MG = mean gradient. Adapted with permission from [27].

**Fig. 7.**
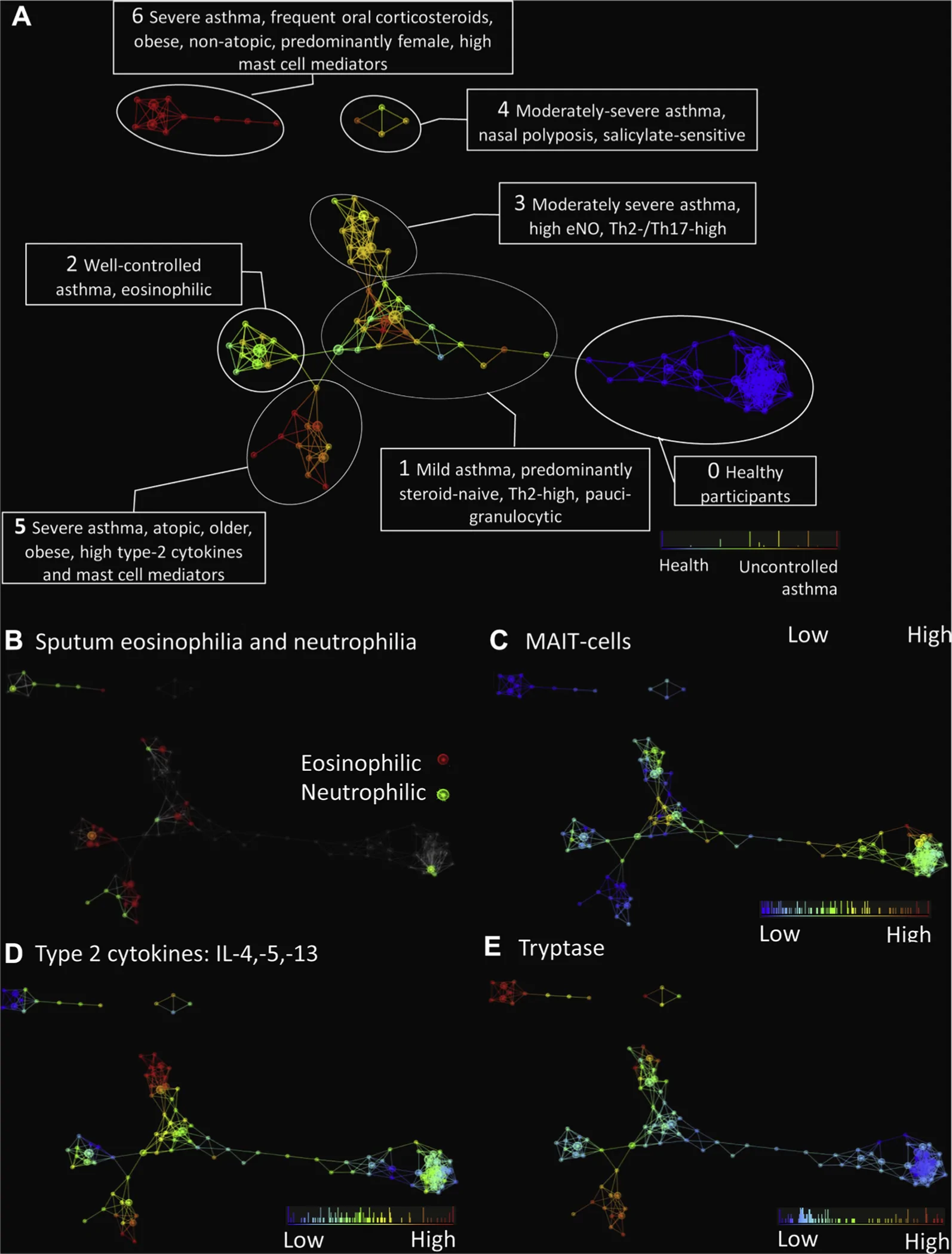
**A.** Multidimensional clinicopathobiologic clusters in asthmatic patients and healthy subjects. Topological network analysis of clinical and pathobiologic features generates 1 healthy *(blue)* and 6 distinct clinicopathobiologic asthma clusters (1–6). The network is colored by disease severity (GINA classification), with patients with the most severe disease in *red* and patients with the milder forms in varying shades of *orange*, *yellow*, and *green*. **B.** The same network as in A, overlaid with distribution of neutrophilic (sputum neutrophils > 61%, *green*) or eosinophilic (sputum eosinophils > 3%, *red*) asthma. **C.** Frequencies of MAIT cells. **D.** The network is colored based on average concentrations of the type 2 cytokines IL-4, IL-5, and IL-13 in serum, sputum, and BAL fluid. **E.** The network is colored based on concentrations of mast cell tryptase in sputum and BAL fluid. In B-E, the colors represent concentrations or frequencies, ranging from low *(blue)* to high *(red)* concentrations. The TDA used 62 subjects with most complete data. The variance normalized Euclidean metric was used. The lenses used were principal and secondary singular value decomposition (resolution, 32; gain, 4.0/3.5× ; equalized). Node size is proportional to the number of subjects in the node. Adapted with permission from [69].

**Fig. 8.**
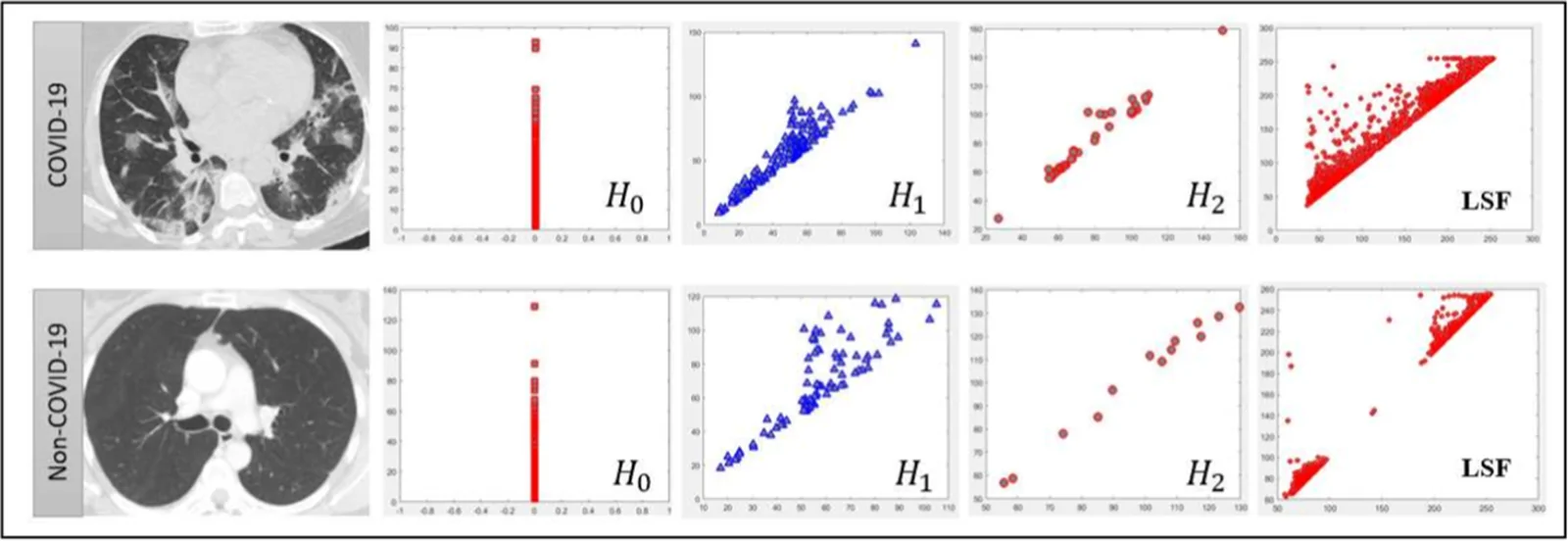
Persistence diagrams of *H*_0_, *H*_1_, *H*_2_, and lower-star-filtration (LSF) of a COVID-19 infected *(top)* and a non-COVID-19 *(bottom)* CT scan. Adapted with permission from [73].

**Fig. 9.**
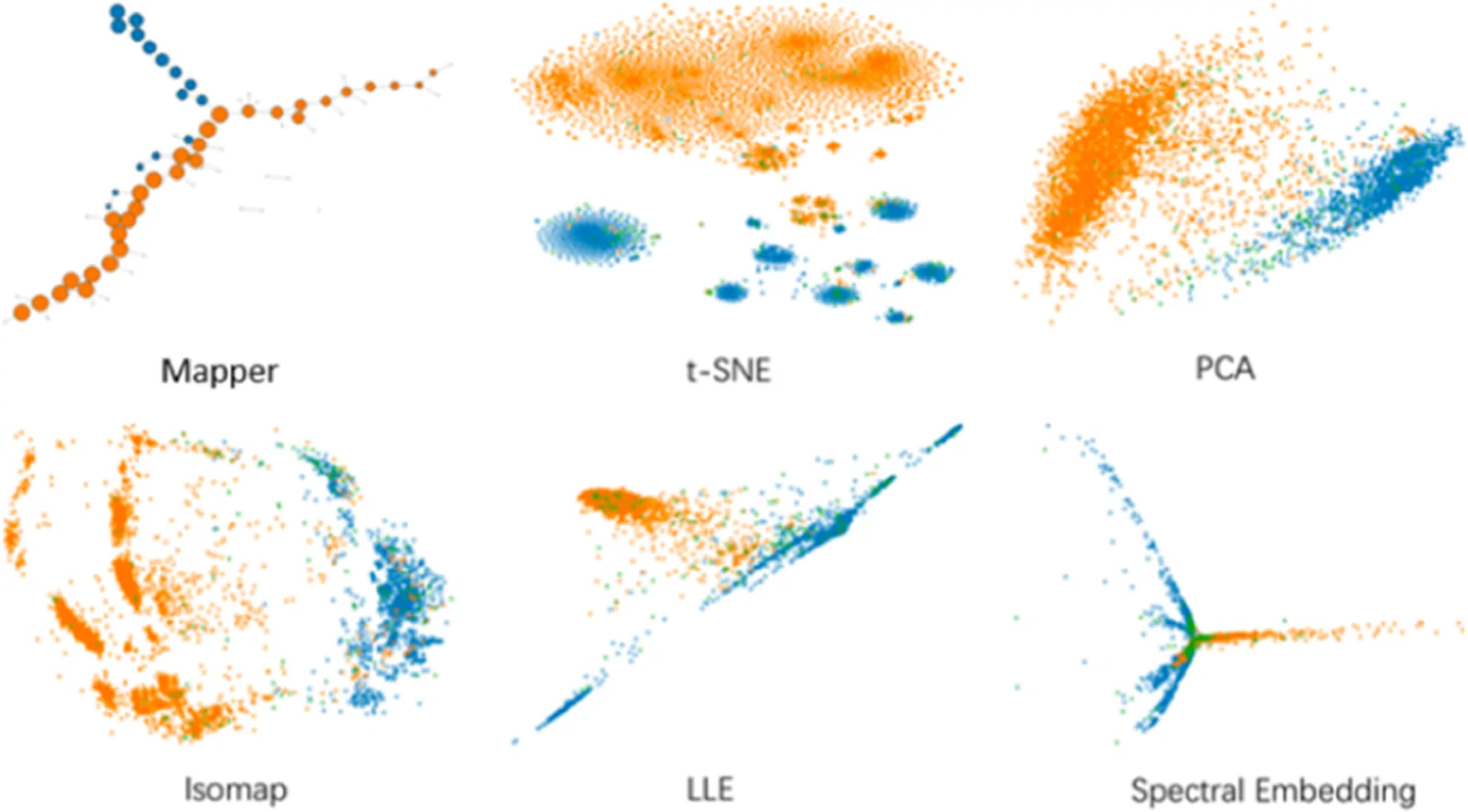
Visualization of scRNA-seq data from melanoma cells (blue = malignant, orange = non-malignant, green = unresolved). Notice how Mapper (top left) distinguishes as many cell types as the other methods, but also depicts a continuum of cell phenotypes along the transition from normal to malignant. Though it depicts the same cell types along with additional information encoded in the edges connecting similar clusters, the Mapper representation is also visually simpler with fewer nodes, thus potentially easier to explore. Adapted with permission from [142].

**Fig. 10.**
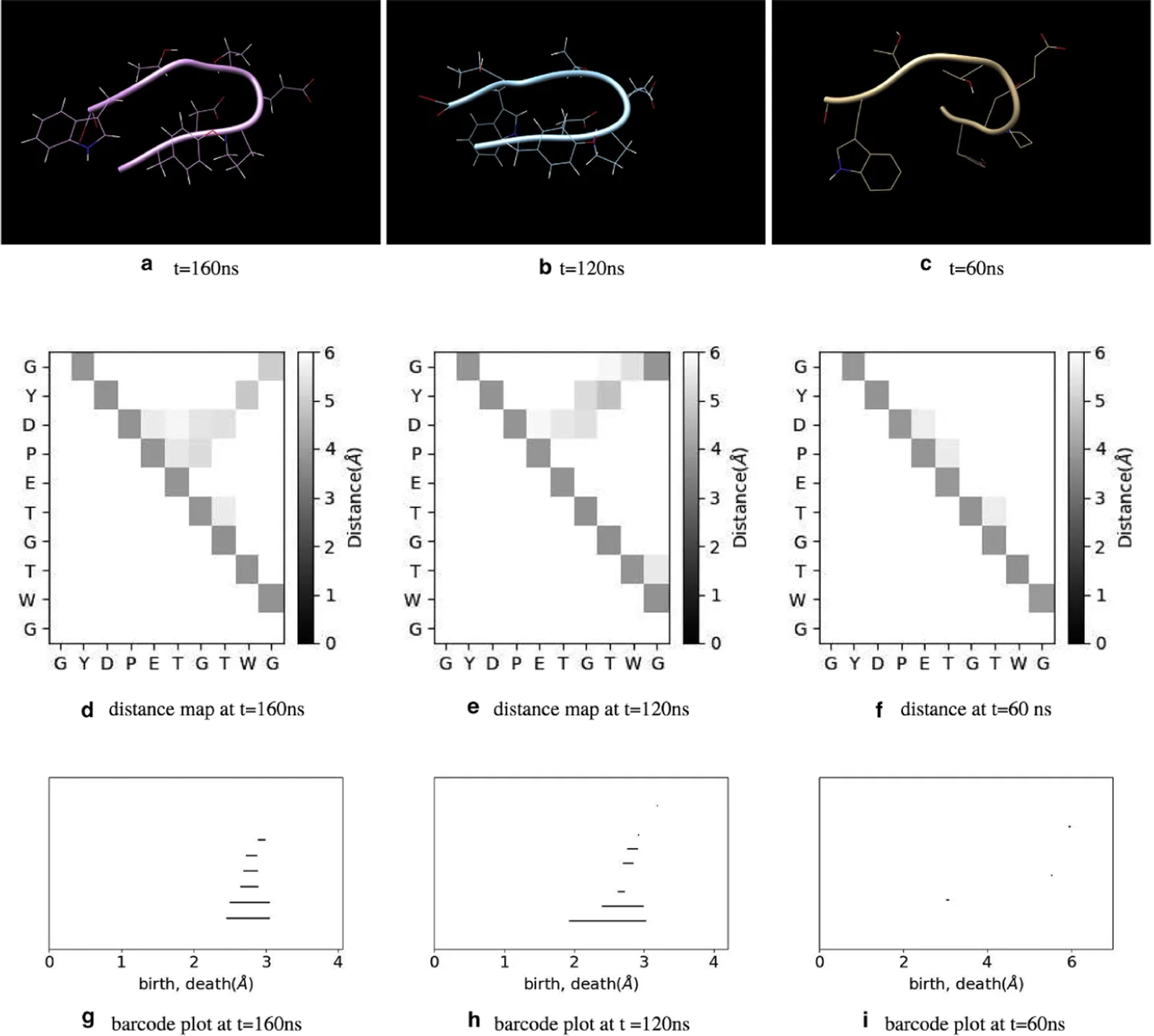
**(a)–(c)** Example of protein configuration in native, misfolded, and unfolded state. **(a)** shows the native state, *t* = 160 ns, (*h*_1_, *h*_2_) = (22.7, 3.0.3). **(b)** shows the misfolded state, *t* = 120 ns, (*h*_1_, *h*_2_) = (3.81,23.1). **(c)** shows the unfolded state, *t* = 60 ns, (*h*1, *h*2) = (3.60, 6.44). **(d)–(f)** show examples of distance maps for native **(d)**, misfolded **(e)**, and unfolded states **(f)**. **(g)–(i)** show persistent barcode plots for native **(g)**, misfolded **(h)**, and unfolded states **(i)**. Adapted with permission from [72].

**Fig. 11.**
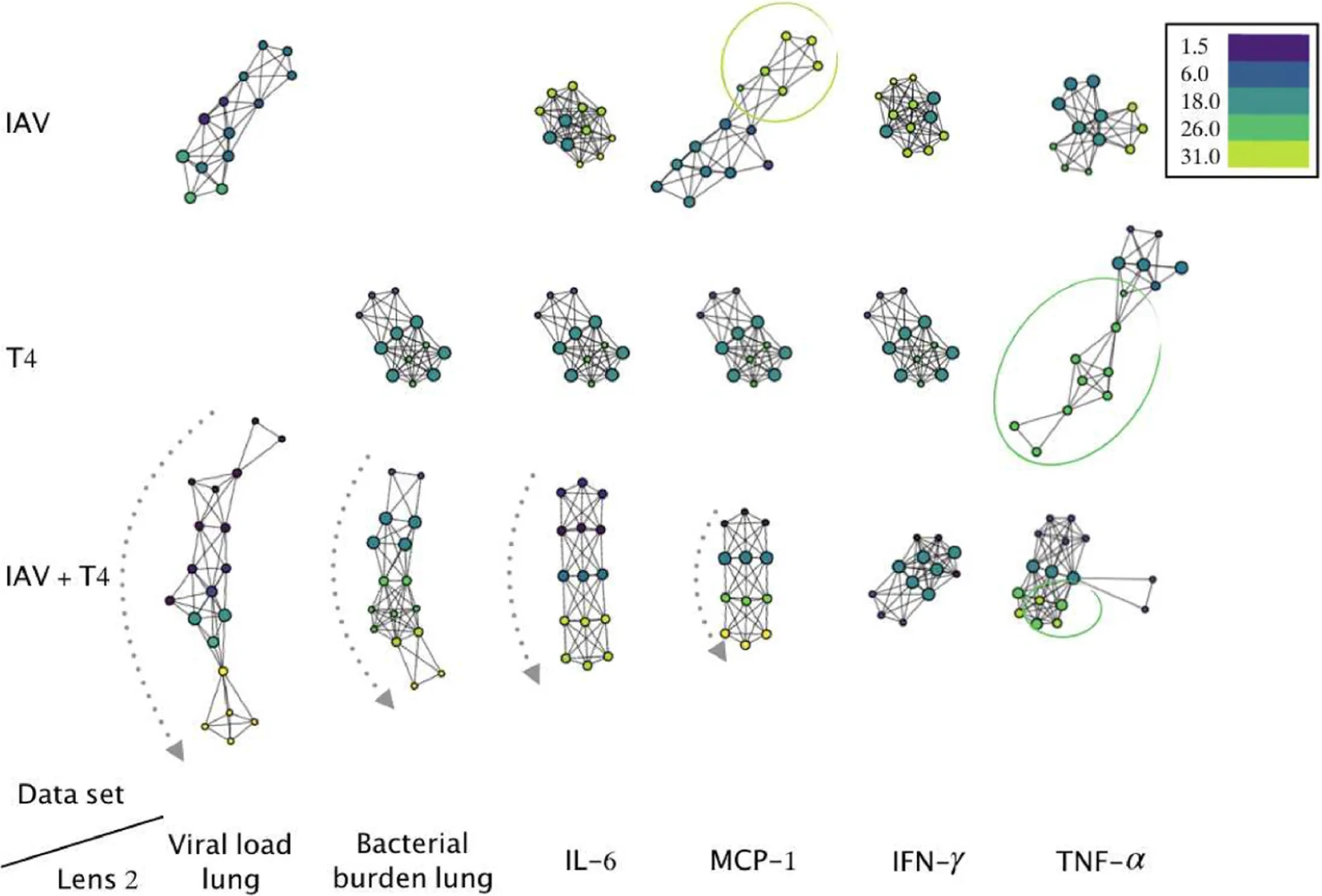
Decoupling simplicial complexes of the immune response to virus, bacteria and co-infection. Five simplicial complexes are generated for the single viral (top row) and single bacterial (middle row) infection groups and six simplicial complexes are generated for the co-infection group (bottom row). The vertices of the simplicial complexes are color (see legends) coded according to the hour post infection or co-infection in the clusters. Clusters that contain data subsets belonging to more than one time point are colored by the average color value for members in that node. Adapted with permission from [131].

**Fig. 12.**
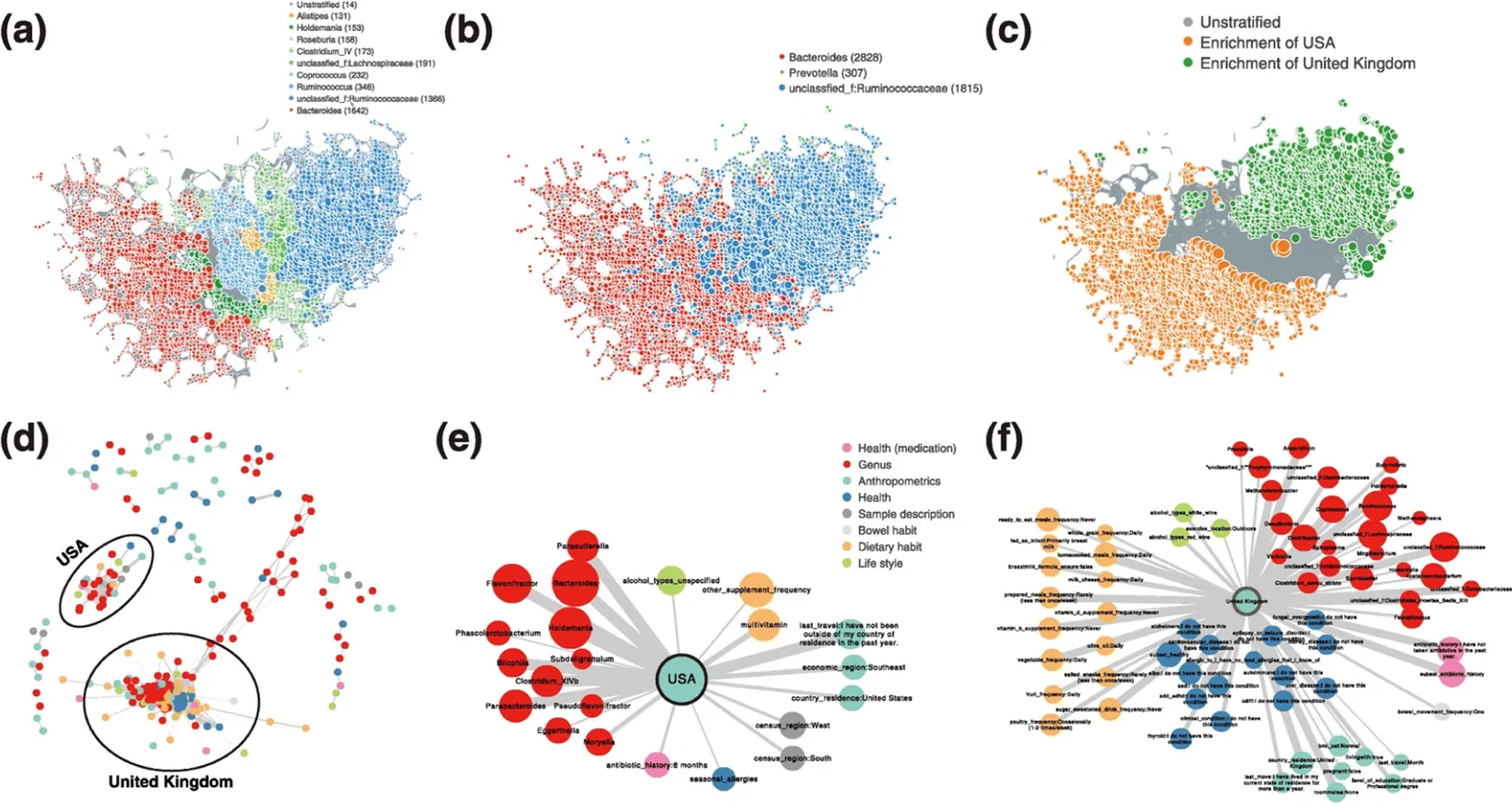
In-depth analysis of enterotype-like stratification of the AGP microbiomes and association with lifestyles. **a** Stratification of the AGP microbiomes based on enriched taxa. For each node in the TDA network, the most enriched taxon among all taxa is identified according to SAFE enriched score. Each node is colored according to its most enriched taxon. Only taxa enriched in more than 100 nodes are highlighted. Remaining unstratified nodes (with no enriched taxa) are colored in gray. **b** Stratification based on traditional enterotype analysis. Nodes are colored according to enterotype driver taxa. **c** Stratification based on countries (USA or UK). Not enriched (or unstratified) nodes are colored in gray. The number in the color legend indicates the number of nodes in the corresponding stratification. **d–f** Co-enrichment networks of lifestyle factors and taxa. Co-enrichments with countries (USA or UK) are highlighted and extracted. The extracted co-enrichment subnetworks reveal that different lifestyle factors are interrelated to the two countries when accounting for the AGP microbiomes. Node colors are based on metadata category. Node size is scaled according to the SAFE enriched score of metadata or taxa. Edge width of the network is scaled according to the negative log-transformed p value of Fisher’s exact test of co-enrichment. Adapted with permission from [88].

**Fig. 13.**
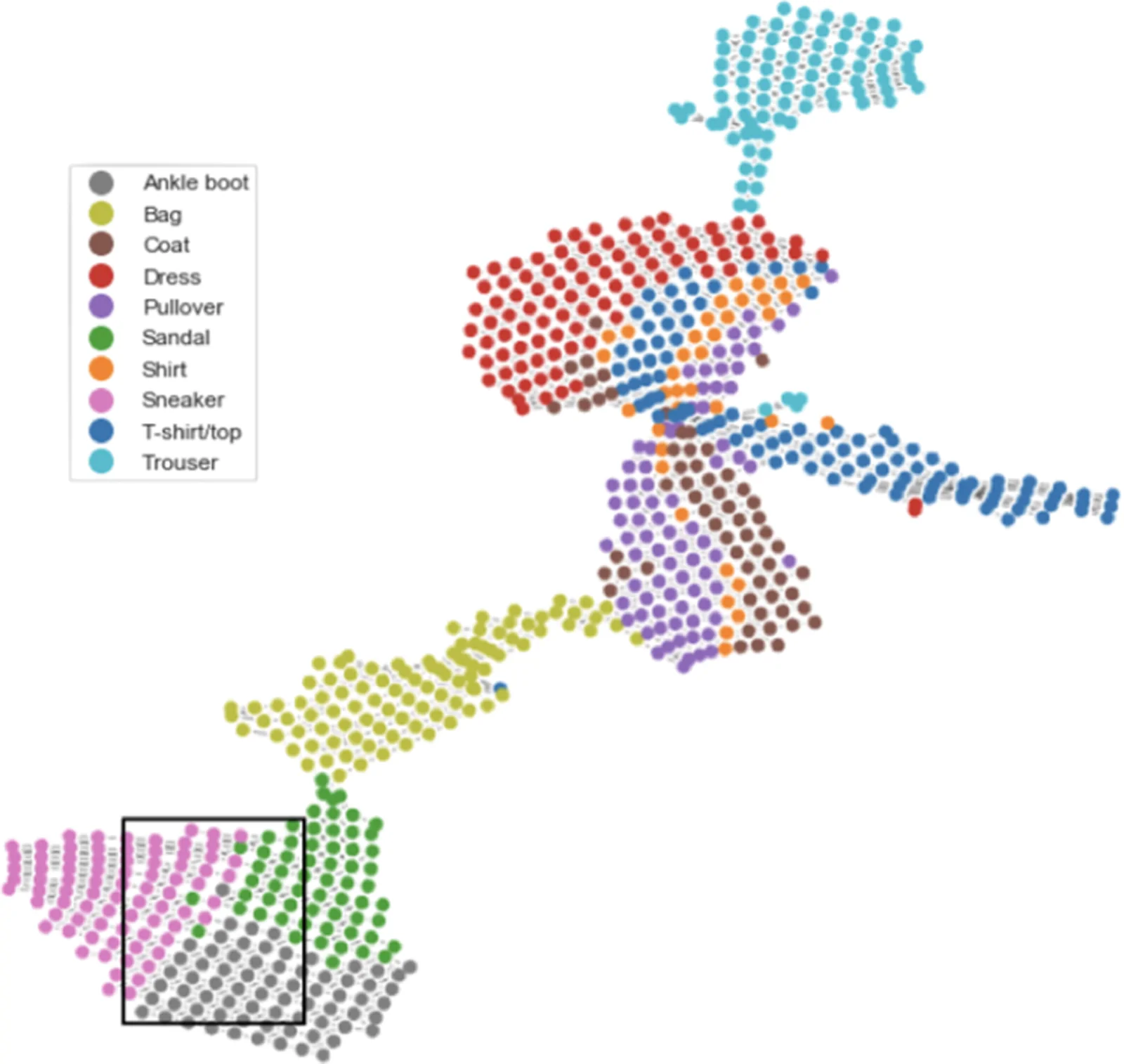
Mapper visualization of the predicted probability space of an LR classifier trained to place images of clothing into one of several categories (shirt, trousers, etc.). Observe that areas comprised of data classified as “bag” or “trousers” are very distinct from the rest of the simplicial complex, indicating these categories of images are easily delineated. On the other hand, “T-shirt”, “shirt”, and “pullover” categories are all mixed together in a central area, insinuating that these categories are more ambiguous and that model has trouble distinguishing them.

**Fig. 14.**
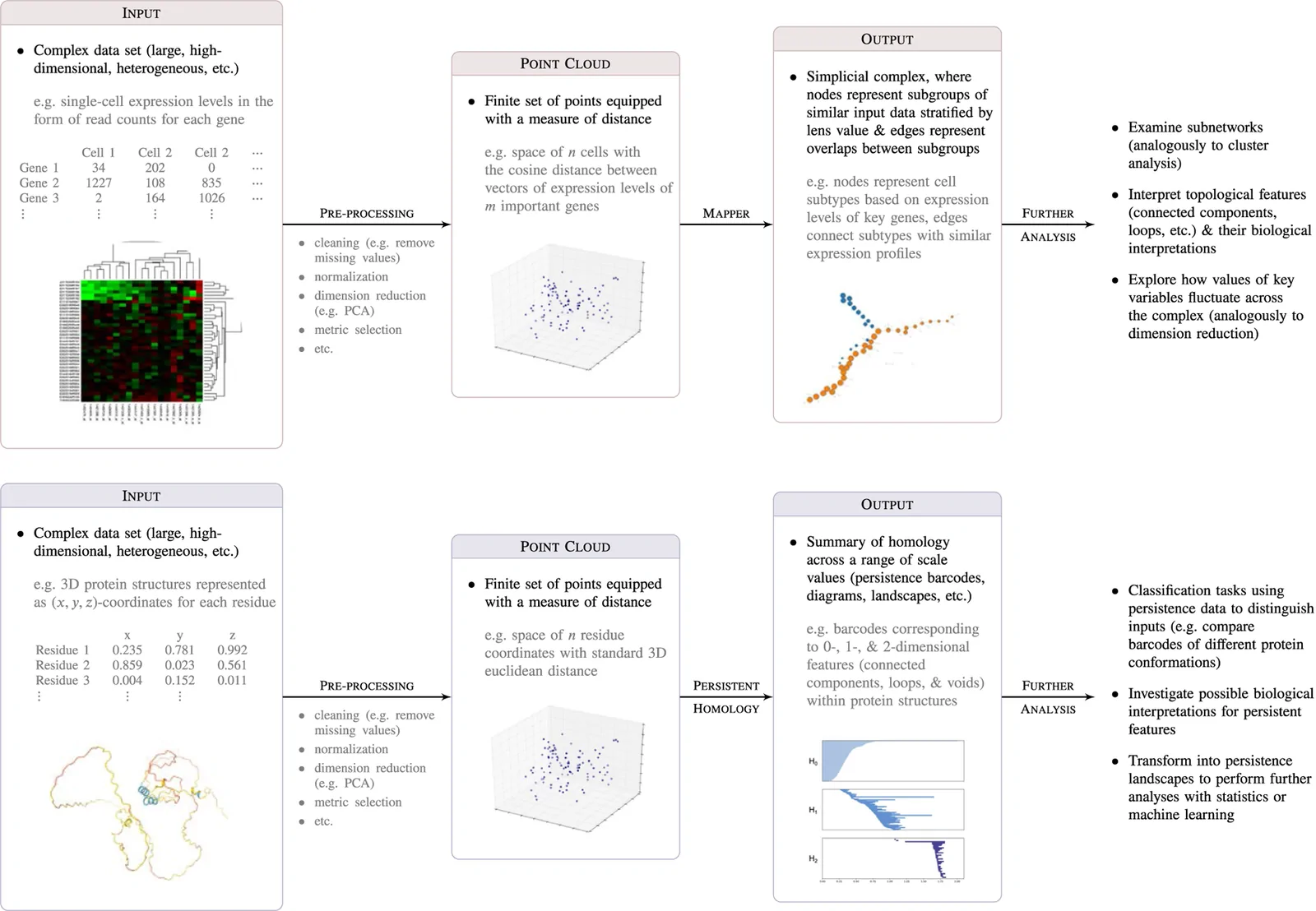
Overview of potential TDA pipelines on example biometical data sets.

**Fig. 15.**
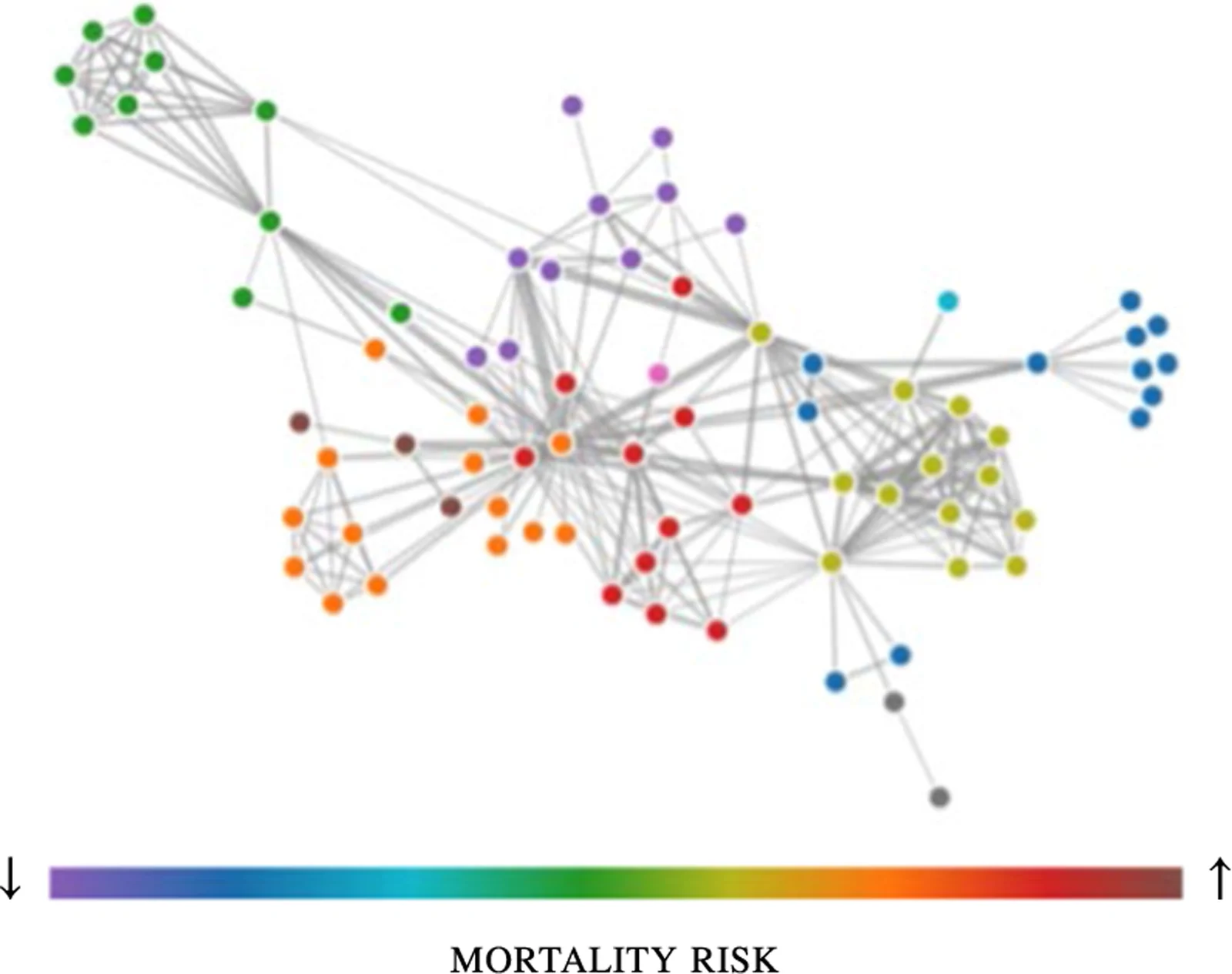
Suppose the network to the left was constructed from a Mapper model using a lens that incorporates values of mortality risk predicted by an ML classifier. The central area of the network contains a mixture of high risk nodes *(orange, red, brown)* that would represent subtypes of patients with high risk phenotypes that are difficult to tease apart due to overlapping features. For example, patients in end-stage heart failure and end-stage renal failure may become difficult to distinguish as they both deteriorate toward multi-system failure, at which point many of their clinical variables will be very similar. Several subnetworks of lower risk nodes *(green, blue, purple)* around the edges of the network appear relatively distinct from the central area, which would indicate these phenotypes are easily dinstinguishable by clinical variables, analogously to how the “bag” subnetwork in Fig. 13 appeared fairly separated from visually dissimilar items such as shirts. Based on this network, we could hypothesize that it might be more effective to attempt to design a treatment plan to modify the clinical characteristics of a patient in one of the orange nodes in the bottom left toward those in the green subnetwork at the top left, as opposed to attempting a longer path that may be riskier and more complex toward the blue subnetwork at the far right.

**Table 1:** Overview of Biomedical Applications of TDA. Abbreviations: PH, persistent homology; T2DM, type 2 diabetes mellitus; TBI, traumatic brain injury; DSGA, disease-specific genome analysis; ER+, estrogen receptor-positive; COPD, chronic obstructive pulmonary disease; OA, osteoarthritis; NN, neural network; PE, pulmonary embolism; scRNA-seq, single cell RNA-sequencing.

Topic	Subtopic	Reference	Method	Application	Data Type	Summary of Contribution

Clinical Medicine	Disease Subtyping	Li et al. [87]	Mapper	T2DM	EHR	Mapper was used to identify novel subtypes of T2DM based on clinical variables.
		Dagliati et al. [45]	Mapper	T2DM	time series	Distinct disease trajectories (temporal phenotypes) of T2DM subtypes were characterized using Mapper.
		Casaclang-Verzosa et al. [27]	Mapper	aortic stenosis	echocardiogram variables	Mapper identified subtypes of aortic stenosis with higher resolution than standard approaches.
		Hinks et al. [69]	Mapper	asthma	clinical variables & levels of inflammatory mediators	Mapper identified six subgroups of asthmatic patients with distinct clinical and pathological characteristics.
		Siddiqui et al. [135]	Mapper	asthma	bronchial biopsy histology variables	Pathological airway changes in asthma are heterogeneous, and Mapper can help identify patient subgroups and characterize transitions between healthy and diseased states.
		Kuo et al. [80]	Mapper	asthma	clinical & bronchial biopsy histology variables	Mapper identified two subtypes of asthma, one with high expression of T_*H*_2 cytokines and the other by decreased corticosteroid response.
		Bigler et al. [13]	Mapper	asthma	transcriptome (blood)	Blood transcriptomics data can be used with Mapper to identify phenotypic subtypes of asthma patients.
		Nielson et al. [107,106]	Mapper	TBI	clinical, genetic, & histology variables	Mapper identified patient subgroups as well as clinical & genetic predictors of long-term recovery after TBI.
		Nicolau et al. [105]	Mapper	breast cancer	transcriptional microarray	Using DSGA as a lens for Mapper identified a novel subtype of ER + breast cancer tumors that was undetected by traditional tools.
	Medical Imaging	Belchi et al. [11]	PH	COPD	CT images	PH on geometric features extracted from chest CT images can classify COPD patients more finely than standard radiographic measurements.
		Iqbal et al. [73]	PH	COVID-19	CT images	Persistence diagrams can distinguish CT scans of COVID-19 patients from healthy controls with equivalent or better accuracy than current gold standard approaches and without a need for pre-classified training data.
		Pedoia et al. [111,125]	Mapper	OA	MRI variables	Using PCA as a lens for Mapper on variables extracted from MRI scans separated OA patients from healthy controls, identified predictors of disease progression, and separated different stages of OA as subnetworks.
		Lawson et al. [83,82]	PH	prostate cancer	histology images	PH was able to recapitulate gold standard grading systems for prostate cancer as well as identify more granular disease subtypes within grades.
		Rojas-Moreleda et al. [123,100]	PH	cell morphology	histology images	PH can detect and classify segmentation of cell nuclei based on histology images.
		Qaiser et al. [117]	PH	cell morphology	histology images	A similar PH-based algorithm to [123,100] was again successful at detecting nuclear segmentation on histology.
		Takiyama et al. [139]	PH	breast cancer	immunohistochemistry	Labelling by PH generated equivalent results to trained pathologists for grading invasive ductal breast carcinoma.
		Duneva et al. [52]	PH	stomach lesions	endoscopy images	TDA, geometric, and ML tools were used in conjunction to classify subtypes of stomach lesions with high accuracy.
	Diagnostics	Rucco et al. [126]	Mapper	PE	angio-CT images, clinical variables	Mapper in conjunction with artificial NNs was able to classify subtypes of both PE and healthy patients. Mapper also identified clinical variables that were most associated with PE, which were then used to improve the accuracy of the NNs.
		Dindin et al. [48]	PH	cardiac arrhythmias	EKG signals	Including a PH-based channel in a multi-channel NN for EKG monitoring reduces computational complexity and outperformed standard methods for arrhythmia detection based on EKG signals.
Biology	Omics	Wang et al. [142]	Mapper	melanoma	scRNA-seq	Mapper can be used to illustrate cellular transitions from healthy to malignant cell types in melanoma samples, which improves upon the cell type classification alone achieved by standard clustering.
		Zechel et al. [147]	Mapper	brain mapping	scRNA-seq	Using PCA as a lens for Mapper on scRNA-seq data that had been spatially mapped back onto images of mouse brain cryosections allowed for characterization of transcriptionally distinct subregions of the brain.
		Setty et al. [134]	Mapper	cellular development	scRNA-seq, mass cytometry	Wishbone, a Mapper-based algorithm, outperformed standard methods at identifying and correctly ordering stages of T-cell development.
		Rizvi et al. [122]	Mapper	cellular development	scRNA-seq	Mapper was used to develop the scTDA algorithm for unsupervised analysis of transcriptional regulation. This method outperformed all others tested in its ability to order cell types along a cellular differentiation process.
		Jeitziner et al. [74]	Mapper	cell subtyping	global gene expression profiles	TTMap is an unbiased, Mapper-based clustering method that can automatically optimize parameters without input from the user and was shown to be effective at detecting cellular subpopulations by their expression profiles. TTMap can also be used to detect subgroups at a smaller scale within subpopulations.
		Zheng et al. [149]	PH	multi-scale omics	scRNA-seq, protein interaction networks	HiDeF is a PH-based method for studying the structure of biological data at multiple scales simultaneously. HiDeF was used to identify known as well as more granular subtypes within mouse scRNA-seq data as well as to analyze protein–protein interaction networks.
		Bowman et al. [18]	Mapper	RNA folding	3D RNA structures	
	Structural Biology					Mapper as used to identify distinct molecular states in the RNA hairpin folding process, providing insight
		Xia et al. [146]	PH	protein structure	3D protein structures	into its mechanism. Molecular topological fingerprints (MTFs) based on PH can be used for protein identification, classification, quantitative analysis of rigidity, and evolution of structural features during folding.
		Swenson et al. [138]	PH	protein structure & function	protein sequences, 3D protein structures	PersGNN combines graph representation learning and PH to capture both local and global structural features. This combined tool outperforms either method in isolation as well as standard NNs for predicting protein function from structural data.
		Kovacev-Nikolik et al. [77]	PH	protein folding	X-ray crystallography	Persistence landscapes are a summary statistic that allow PH to be used in conjunction with statistical and ML models. Landscapes were used to distinguish open and closed conformations of maltose-bi nding protein (MBP) and characterize ligand binding based on X-ray crystallography structures.
		Ichinomiya et al. [72]	PH	protein folding	molecular dynamics	Topological feature vectors (TFVs) comprised of PH data were used as input for PCA to demonstrate several distinct structural states in the folding process of the protein chignolin.
	Immunology	Sasaki et al. [131]	Mapper	immune response to co-infection	immunological variables (e.g. cytokine levels, viral load)	Mapper was used to visualize high-dimensional immunological data pertaining to co-infection of mice with influenza and pneumococcus, allowing for characterization of distinct immune responses in various infection scenarios and stages within immune responses to co-infection.
		Liao et al. [88]	Mapper	microbiomes	microbiome profile data	A Mapper-based algorithm called tmap was used to identify nonlinear features of microbiome data that were not detected by other tools, identify nonlinear and multiscale relationships in these data, and stratify microbiome profiles.
Public Health	Epidemiology	Taylor et al. [141]	PH	contagion modeling	contagion networks	PH can be used with nonlinear dimension reduction can be used to study the spread of contagions across a network from geometric and topological perspectives.
	Healthcare	Dugast et al. [51]	PH	patient flow analysis	hospital admission data	A PH-based method outperformed standard algorithms for detecting increases in ED patient admissions resulting from epidemic events.

**Table 2:** Summary of TDA methods & their applications.

Method	Input	Output	Major Uses	Common Types of Input Data	Biomedical Applications	
					Domain	Example Results

Mapper	point cloud data	simplicial complex			Disease subtyping	Diabetes [87,45], aortic stenosis [27], asthma [69,135,80,13], traumatic brain injury [107,106], breast cancer [105]
			• Data visualization	• Clinical variables (e. g. EHR data)	Diagnosis & staging	Arthritis [111,125], pulmonary embolism [126]
			• Exploratory data analysis	• Omics data (e.g. scRNA-seq)	Omics	Cell type classification by scRNA-seq [142,147,134,122], global gene expression analysis [74]
			• Hypothesis generation	• Population-level data (e.g. microbiome profiles)	Neuroscience	Brain mapping [147]
					Immunology	Immune responses to co-infections [131], population-level microbiome characterization [88]
Persistent Homology	point cloud data	summary of PH (persistence barcodes, diagrams, landscapes, etc.)	• Quantitative data summary	• Images (e.g. CT scans)	Image analysis Analysis of time series data	CT [11,73], histology [83,82,123,100,117,139], endoscopy [52] Arrhythmia detection [48], patient flow analysis [51]
			• Topological fingerprinting	• Time series (e.g. patient flow)	Multi-scale data analysis	Multi-scale omics [149]
			• Multiscale modeling	• Structural data (e.g. 3D molecular structures)	Macromolecule structure & function	Protein structure [146], protein structure–function relationships [138], protein folding [77,72]
					Network dynamics	Contagion modeling [141]

## Footnotes

1 TDA also includes nonlinear dimension reduction (NLDR) by some definitions, but we forgo discussion of such methods here.
